# An Overview of Avian Vaccination Options in Zoological Collections in Europe

**DOI:** 10.3390/vetsci13020148

**Published:** 2026-02-04

**Authors:** Jonas Leus, Margot Morel, Hermann Kempf, Francis Vercammen, Remco A. Nederlof, Jaco Bakker

**Affiliations:** 1 DAP Galluvet nv, 3560 Lummen, Belgium; jonas@galluvet.be; 2 Broadway Veterinary Group, Unit 1 the Links, Herne CT6 7FE, UK; morelmargot2@hotmail.fr; 3 Tierärztliche Praxis fur Exoten, 86167 Augsburg, Germany; hermann.kempf@gmx.de; 4 Centre for Research and Conservation, Royal Zoological Society of Antwerp, 2018 Antwerp, Belgium; francis.vercammen@kmda.org; 5 Independent Researcher, 2861 XZ Bergambacht, The Netherlands; remco.a.nederlof@gmail.com; 6 Animal Science Department, Biomedical Primate Research Centre, 2288 GJ Rijswijk, The Netherlands

**Keywords:** birds, vaccination, zoo, zoonosis, one health, preventive medicine, prophylaxis, biosecurity, autogenous vaccines

## Abstract

Captive bird populations in zoological institutions face unique disease risks due to close interspecies contact and human interaction. Vaccination is widely used as a prophylactic measure. However, most available vaccines are developed for poultry and are used off-label in non-domesticated birds, often without species-specific safety and efficacy data. This review provides a comprehensive overview of vaccines reported in zoo-housed birds for major viral, bacterial, and fungal pathogens. This review highlights that for most vaccines, evidence of safety and effectiveness is limited. Vaccine use should therefore be guided by risk assessment, relevant legislation, and institutional priorities, and should integrate species-specific data on vaccine safety and effectiveness, disease susceptibility, and local epidemiology. Extensive research and species-specific validation are essential to improve preventive health strategies in avian conservation programs.

## Simple Summary

Vaccination can help protect zoo birds from many infectious diseases, but most available avian vaccines are developed for poultry rather than for the diverse species kept in zoological institutions. As a result, veterinarians often use off-label vaccines, even though there is limited evidence of their safety and effectiveness in non-domesticated birds. This review summarizes all published information on vaccines used or suitable for use in zoo-housed birds. Although many commercial vaccines are safely used in poultry, data about their use in non-domesticated birds are scarce, and immune responses may vary greatly between avian species. Good biosecurity, careful risk assessment, and species-specific considerations remain essential. More research is urgently needed to support evidence-based vaccination strategies for endangered and valuable bird species housed in zoos.

## 1. Introduction

From a welfare and epidemiological perspective, zoological institutions present unique challenges [1,2,3,4]. Direct and indirect contact between animals that would not naturally interact in the wild frequently occurs, and animals frequently come into direct or indirect contact with human caretakers and zoo visitors. Although appropriate hygiene and biosafety measures should always be implemented, vaccination of susceptible animals is an additional prophylactic measure to safeguard the health of animals. Vaccination may also contribute to public health, as some pathogens may be potentially zoonotic.

Despite their utility in preventive medicine, vaccination protocols remain scarcely reported in non-domestic avian species [5,6,7]. If used, vaccines are generally administered off-label, and vaccination protocols differ per institution in accordance with local and national veterinary legislation and the discretion of the attending veterinarian.

This paper aims to not only address the paucity of the literature surrounding the use and effectiveness of vaccines in birds, but also to provide veterinary clinicians working with birds with an overview of current peer-reviewed evidence on the different available vaccines. The manuscript is intended to assist veterinary professionals in the construction of their institution-specific immunization and biosafety protocols.

## 2. Methodology

To identify the relevant literature, a search was conducted for publications in academic literature databases, such as PubMed, Scopus, and Web of Science. Subtopic-specific word combinations were used, including “vaccination”, “zoo”, “avian”, and “birds”. Reports were subsequently critically evaluated for relevance. Rejected manuscripts included those where the full manuscript was not available, those not written in English, or manuscripts that were insufficiently comprehensive for comparison with other studies. Experimental vaccines were included in this review when they provided relevant safety, immunogenicity, or efficacy data for non-domesticated birds. Serological studies assessing antibody responses without clinical endpoints were interpreted as indicators of immunogenicity rather than protection. Regarding commercial poultry vaccines, peer-reviewed evidence is provided if they are available; otherwise, leaflet recommendations are provided. Expert opinion and case reports were used to contextualize practical application but were not considered confirmatory evidence of efficacy [8,9,10].

Evidence supporting vaccine safety, immunogenicity, and protective efficacy in zoo birds is frequently extrapolated from poultry studies, with limited species-specific data available. This necessitates cautious interpretation of vaccine outcomes, particularly where conclusions are based on serological responses rather than clinical protection [11,12]. Vaccine-induced antibody responses are frequently reported in studies involving non-domesticated avian species. However, seroconversion alone does not reliably predict clinical protection, particularly in the absence of challenge studies or documented reductions in morbidity or mortality. Therefore, studies reporting serological responses without clinical endpoints should be interpreted as evidence of immunogenicity rather than confirmed protective efficacy [8,13].

## 3. Viral Etiology

### 3.1. Avipoxvirus

**Figure 1:**
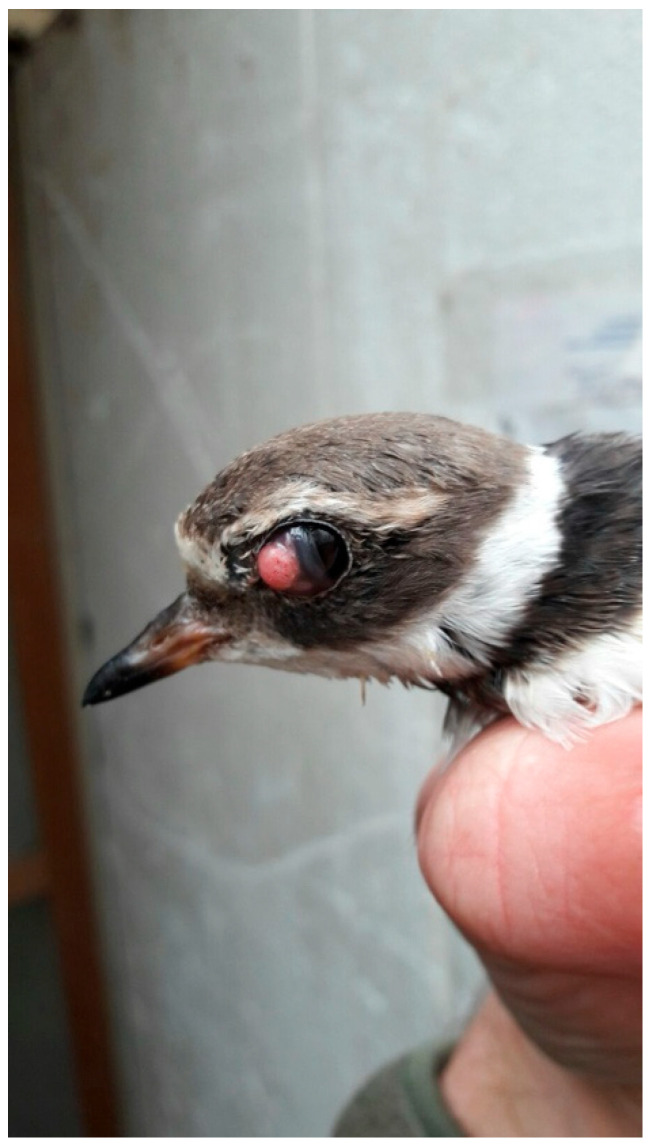
Common ringed plover (*Charadrius hiaticula*) with an APV infection on the third eyelid (photograph provided by H. Kempf).

*Avipoxvirus* (APV) is a genus of poxviruses frequently affecting poultry, pets, and different wild birds [14]. The genus *Avipoxvirus* contains 12 recognized species based on disease characteristics, host and ecological niche, and growth characteristics on cellular culture. The phylogenetic reconstruction identifies at least three highly divergent major genetic lineages: Clade A (which includes *Avipoxvirus fowlpox* (FP)); Clade B, including *Avipoxvirus canarypox*; and Clade C, which is associated with psittacine poxviruses [15,16,17,18,19]. There are two unique manifestations of the disease: the cutaneous nodular (dry), which is associated with a low mortality rate, and nodular skin lesions, which are mostly found on the eyelids, combs, and thighs [6,17,20,21,22]. Clinical signs associated with APV infection include anorexia, weight loss, stunted growth, alopecia of feathered skins with yellow scabs, respiratory distress, and depression. Due to involvement of the eyes, some affected birds demonstrate unilateral or bilateral blindness secondary to blepharitis and conjunctivitis (Figure 1). APV infection is not generally considered to be the direct cause of mortality of all affected birds, but the lesions that this disease produces may increase the risk of accident, predation, and secondary infection, and may reduce breeding and foraging success [21].

#### 3.1.1. *Avipoxvirus* Clade A

**Figure 2:**
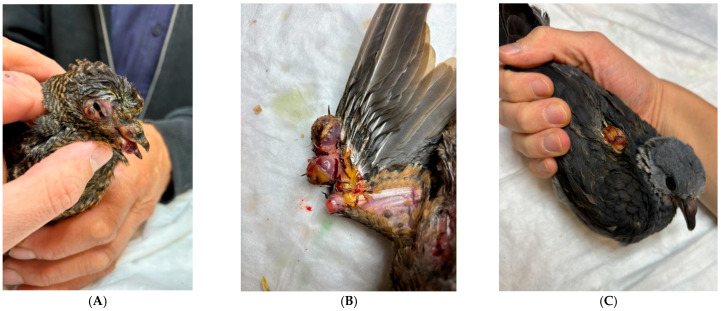
APV infection in different species of non-domesticated pigeons. (**A**) Barred cuckoo-dove (*Macropygia unchall*) with pox lesions on eyelids, nose, and beak; (**B**) Stephan’s emerald dove (*Chalcophaps stephani*) with pox lesions on the wing; (**C**) Ashy wood pigeon (*Columba pulchricollis*) with pox lesions on the wing (photographs provided by J. Leus).

In domestic pigeons (*Columba livia*), pox lesions are caused by *Avipoxvirus pigeonpox*. Other Columbiformes may be similarly affected, including the barred cuckoo-dove (*Macropygia unchall*) (Figure 2A), Stephan’s emerald dove (*Chalcophaps stephani*) (Figure 2B), ashy wood pigeon (*C. pulchricollis*) (Figure 2C), mourning dove (*Zenaida macroura*), and the Socorro dove (*Z. graysoni*) [23,24].

Vectormune^®^ FP ILT (CEVA-Phylaxia Co., Ltd., 1107 Budapest, Hungary) and Vectormune^®^ FP ILT + AE (CEVA-Phylaxia Co., Ltd., 1107 Budapest, Hungary) are live vector freeze-dried wing web vaccines against FP, infectious laryngotracheitis (ILT), and avian encephalomyelitis (AE). The leaflet recommends administering the vaccine to susceptible chickens (*Gallus gallus domesticus*) of 8 to 13 weeks of age. Chickens receiving this vaccine must not have been previously exposed to field FP virus or pox vaccine due to interference with immunity following vaccination. The onset of immunity starts three weeks (FP, ILT) or 20 weeks (AE) after vaccination. The duration of immunity for FP is 34 weeks after vaccination, and 57 weeks for ILT and AE. There is no information on the use of the vaccine in other birds than chickens.

Diftosec^®^ (Boehringer Ingelheim Animal Health France SCS, 69007 Lyon, France) is a live attenuated FP virus vaccine marketed for wing web application. It was used in Brant geese (*Branta bernicla*) and domestic ducks (*Anas platyrhynchos*) without any reported adverse effects (Leus, pers. comm). No data on vaccine safety and effectiveness are available in non-domesticated bird species.

Nobilis^®^ AE + PD (Intervet Nederland B.V, 5831 AN Boxmeer, The Netherlands) is a live vaccine recommended for wing web application for FP and AE for chickens and turkeys (*Meleagris gallopavo*). It was used in Japanese quail (*Coturnix japonica*), Brant geese, and long-tailed duck (*Clangula hyemalis*) without any reported adverse effects (Leus, pers. comm).

Diftovac^®^ (Pharmagal-Bio spol. S.r.o, 949 01 Nitra, Slovak Republic) can be administered as a wing web or as a subcutaneous (SC) injection. The first administration can be performed at the age of four weeks. Immunity onset will be at 21 days post-vaccination and will persist for at least nine months. Annual booster vaccination is recommended by the manufacturer for commercial poultry. The booster interval may be shortened to nine months if infection pressure is high.

In experimentally infected zebra finches (*Taeniopygia guttata*) (*n* = 25), a commercial FP vaccine (Poxine^®^; Duphar, Fort Dodge, IA, USA) was demonstrated to be effective in preventing clinical disease after inoculating with a silvereye isolate or a blackbird isolate [25]. In Europe, currently, Poxine^®^, AE-poxine^®^ (Zoetis Belgium S.A, 1930 Zaventem, Belgium), and Poxine-AE^®^ (Boehringer Ingelheim Animal Health France SCS, 69007 Lyon, France) are registered for chickens. Both vaccines are recommended for the prevention of AE and FP, and the manufacturer’s advice is to administer them by wing web stab from eight weeks of age.

Pox infections were recorded in ostrich chicks (*Struthio camelus*) [26]. During the outbreak, vaccination was initiated using a commercial FP vaccine (Vineland Labs Ltd., Vineland, ON, Canada) on ostrich chicks between the ages of 10 and 14 days. Pox morbidity immediately decreased dramatically. Subsequently, ostrich chicks were routinely inoculated with this commercial FP vaccine. No APV infections have been observed since this flock.

*Avipoxvirus* also affects birds of prey (order *Falconiformes*). Pigeonpox vaccines and turkeypox vaccines are used in falcons with variable degrees of success [27]. An attenuated *Avipoxvirus* vaccine for *Falconiformes* is available in the Middle East (Falconpox Vaccine^®^, Center Veterinary Research Laboratory, Dubai, United Arab Emirates (UAE)) and has been used successfully for several years. It is recommended to administer the vaccine SC initially at six to nine weeks of age and a booster four weeks later [28,29,30,31,32].

In the Middle East, houbara bustards (*Chlamydotis undulata undulata*) are routinely vaccinated against bustardpox [33,34,35]. A licensed Houbarapox vaccine^®^ (Center Veterinary Research Laboratory, Dubai, UAE) is locally available. 

Morbidity and mortality continued to be reported despite vaccination with a live *Avipoxvirus canarypox*-like strain (Poulvac P Canary; Pfizer, 2909 LD Capelle aan den IJssel, The Netherlands) administered via the wing web route at 30 and 60 days of age, followed by annual boosters [34]. The efficacy of vaccination remains uncertain due to the substantial genetic diversity of strains infecting houbara bustards and the limited knowledge regarding cross-protection among APVs. Further research is needed to elucidate the determinants of host specificity and pathogenicity across APV strains.

The use of Poulvac^®^ Canary Pox FOI (Zoetis Italia S.r.l., 00192 Rome, Italy) has been described to be used in houbara bustards by wing web application at 30 and 60 days, and subsequent annual boosters are provided [36,37]. No adverse effects were observed. Unfortunately, no efficacy information is available.

Commercial FP vaccines may provide safe and appropriate protection in passerine birds exposed to wild APV strains with very high genetic homology to FP [25,26]. However, they should be used with caution due to the lack of controlled safety and efficacy trials in non-domesticated birds.

#### 3.1.2. *Avipoxvirus* Clade B

Poulvac^®^ P Canary (Zoetis Belgium SA, 1348 Louvain-la-Neuve, Belgium) is a freeze-dried vaccine containing the KP1 *Avipoxvirus canarypox* strain and is administered via wing web. The administration is registered in canaries (*Serinus canaria*) and finches. From mid-June to mid-July, canaries should be vaccinated. Later-born offspring must be vaccinated separately, from the age of four weeks. After successful vaccination, a small jar the size of a grain of rice can be observed at the vaccination site.

Experimental administration of a commercial *Avipoxvirus canarypox* vaccine (Biomune Poximmune C^®^, Ceva Biomune, Lenexa, KS, USA) in Hawaiian honeycreepers (*Hawaiʻi ʻamakihi Chlorodrepanis virens*) resulted in life-threatening disseminated lesions or lesions of unusually long duration. After healing of the lesions, the birds were challenged with wild Hawaiian isolates. Protection was demonstrated to be limited. Based on these findings, the manufacturer recommended against the use of this vaccine in honeycreepers [38]. We argue that similar caution should be exercised in other non-domesticated bird species in the absence of safety data.

#### 3.1.3. *Avipoxvirus* Clade C

*Avipoxviruses* have been reported in several psittacine species, including budgerigar (*Melopsittacus undulatus*), white-fronted amazon parrots (*Amazona albifrons*), and love birds (*Agapornis personata* and *A. roseicollis*) [39]. Clinical signs included dying with severe diphtheritic oral, esophageal, and crop lesions.

Peach-faced love birds (*n* = 15), all aged under ten weeks, were immunized by wing web with an experimental vaccine and subsequently challenged with isolates of psittacine poxvirus obtained from imported birds [40]. Results were promising, and the data from this experiment should encourage further trials under field conditions with larger numbers of birds. However, no follow-up studies have been conducted to date to confirm the vaccine’s safety and effectiveness.

### 3.2. Herpesviruses

A large variety of different herpesviruses can induce various disease manifestations in many domestic and wild avian species throughout the world. Disease has been reported in pigeons, psittacines, falcons, owls, cormorants, cranes, storks, and bobwhite quail [41,42].

#### 3.2.1. Pacheco’s Disease

Pacheco’s disease, caused by *Iltovirus psittacidalpha 1* (formerly *Psittacid alphaherpesvirus 1*; PsHV-1), is an acute fulminating hepatitis resulting in mortality in psittacines [43,44,45,46].

Several experimental vaccines have been developed [44,47,48]. Theoretically, live-attenuated herpesvirus vaccines, like those used for Marek’s disease (MD) and ILT, should also be effective in psittacines [47]. However, vaccination of budgerigars with a commercial lyophilized MD vaccine did not prevent infection [43].

The successful use of an autogenous formalin-activated vaccine injected intramuscularly (IM) with one month interval was described in a large collection of psittacines during a Pacheco’s disease outbreak [49]. Another single-dose, autogenous vaccine has also been successfully utilized during an outbreak in different macaws and cockatoos [47].

The authors could not find any vaccines currently commercially available in Europe. Therefore, in case of an outbreak, the use of autogenous vaccines appears to be the best option.

#### 3.2.2. Marek’s Disease

**Figure 3:**
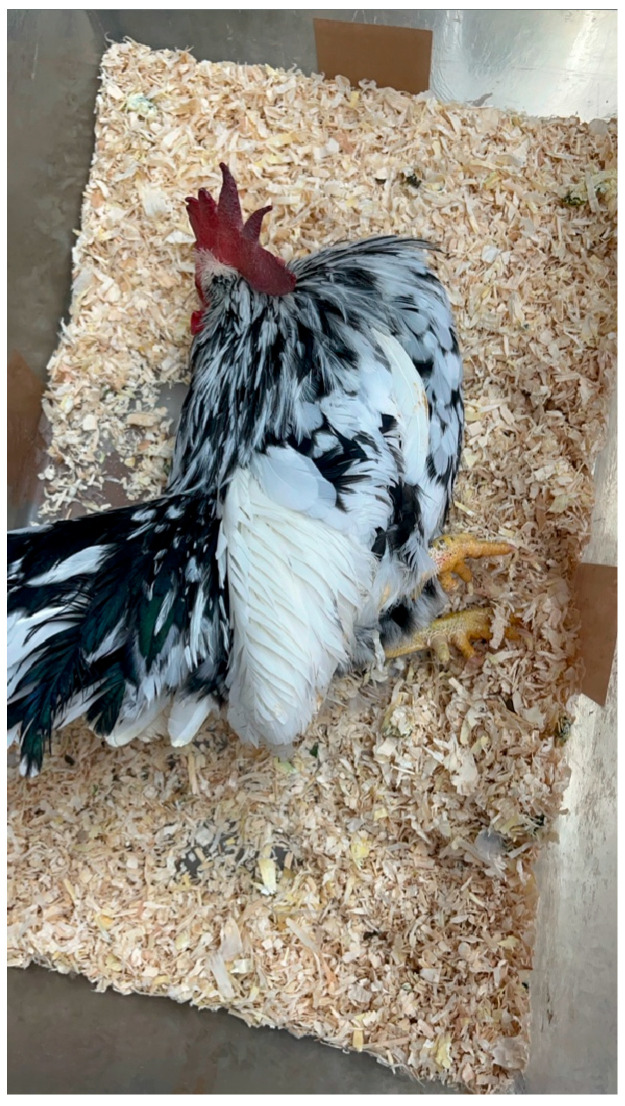
Marek’s disease in a young cock (*Gallus gallus domesticus*) with pathognomonic lameness (photograph provided by H. Kempf).

**Figure 4:**
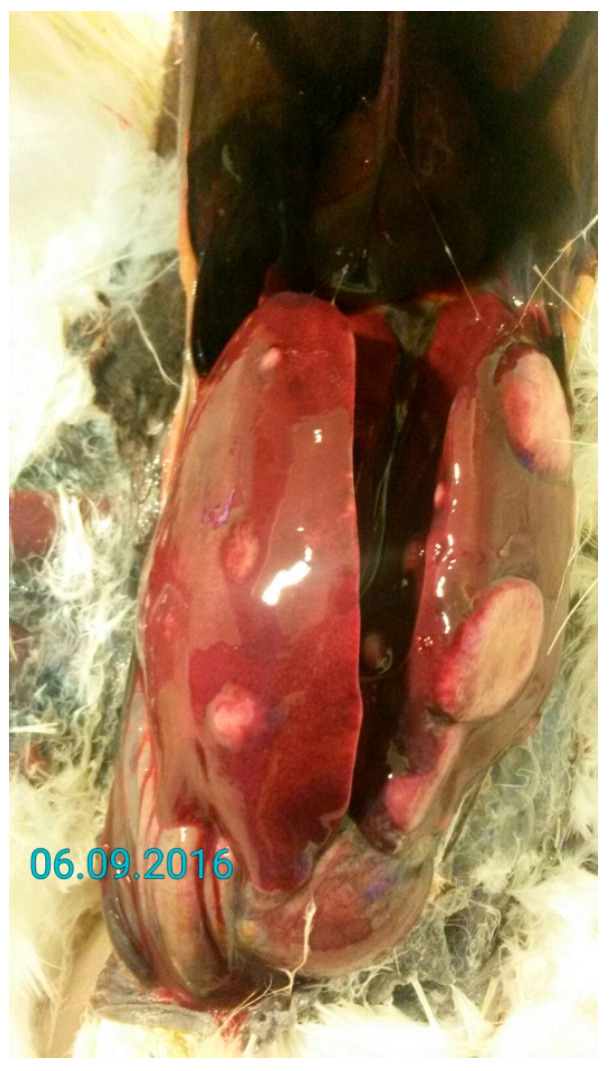
Liver of a chicken (*Gallus gallus domesticus*) with Marek’s disease. Several typical Marek’s disease-associated tumor lesions on the liver are visible (photograph provided by H. Kempf).

MD is a lymphoproliferative disorder caused by *Mardivirus gallidalpha 2* (formerly known as *Gallid alphaherpesvirus 2*; GaHV-2), which mainly affects domestic gallinaceous birds. Sporadic infections in Indian peafowl (*Pavo cristatus*), crested-partridges (*Rollulus roulroul*), Japanese quail, red-crowned cranes (*Grus japonensis*), and domestic and wild ducks (family *Anatidae*) have been reported [50,51,52,53,54,55,56,57]. Since MD may affect any organ system of the body, reported clinical signs vary. Paralysis of the wings and legs, depression, and emaciation are commonly observed (Figure 3). A gray eye with an irregular pupil size is a typical feature of the ocular form of MD. Feather follicles of the skin from the leg and breast region are swollen [6,42,58]. On necropsy, tumors of various visceral organs, skin, or muscle may be observed (Figure 4) [6].

Multiple vaccines are commercially available for MD in poultry. Vaccination of chickens is often performed with a combination of different vaccines. *Mardivirus meleagridalpha 1* (formerly *Meleagrid herpesvirus 1*; MeHV-1), more commonly known as Turkey Herpesvirus (HVT), vaccines are often combined with a Rispens vaccine for a higher and earlier protection against MD [59].

Innovax^®^-ILT-IBD (Intervet International B.V., 5831 AN Boxmeer, The Netherlands) is a live HVT vector vaccine licensed for use in poultry and protects against infectious bursal disease (IBD), ILT, and MD. The vaccine should be administered by SC injection in the neck.

Nobilis^®^ Rismavac (Intervet International B.V., 5831 AN Boxmeer, The Netherlands) is a live vaccine containing a Rispens CVI988 strain. The vaccine should be injected SC in the neck or IM in the leg.

Poulvac^®^ Marek CVI+HVT (Rispens-HVT) (Zoetis Belgium S.A., 1930 Zaventum, Belgium) is a bivalent live virus vaccine. The vaccine should be administered SC.

A combination of Innovax^®^-ILT-IBD and Nobilis^®^ Rismavac has been administered to domestic ducks, turkeys, ring-necked pheasants (*Phasianus colchicus*), Indian peafowl, and Japanese quail with no adverse effects (Leus, pers. comm). The effectiveness of this vaccine combination in other species than chickens has not been formally assessed.

In mountain peacock pheasant (*Polyplectron inopinatum*), Malayan peacock pheasant (*P. malacense*), and Congo peafowl (*Afropavo congensis*), a novel herpesvirus was discovered that is closely related to *Mardivirus gallidalpha 3*. Consequently, caution should be exercised when using MD vaccines in Galliformes other than chickens. The vaccines often include *Mardivirus gallidalpha 3*, HVT, and attenuated MD, and the susceptibility of non-domesticated species to vaccine-*Mardivirus gallidalpha 3* is yet unknown and warrants further research [60,61].

#### 3.2.3. Infectious Laryngotracheitis Virus

*Iltovirus gallidalpha 1* (formerly *Gallid herpesvirus 1*; GaHV-1), or ILT virus, causes disease in all classes of birds, including a range of Galliformes species [62,63,64]. Clinical signs vary from hemorrhagic conjunctivitis, watery eyes, and nasal discharge to respiratory difficulties, including rales, gasping, and expulsion of blood-stained mucus. There may be a sudden onset of rapidly spreading clinical signs and high mortality exceeding 50% [6,42,65,66,67]. The disease severity is assumed to be influenced by the virulence of the specific viral strain, stressors, co-infections with other pathogens, immune status of the flock, and age of the birds [68].

Several types of vaccines are available, including killed, live-attenuated, and recombinant vaccines [69,70,71,72]. Under field conditions, modified live vaccine strains propagated in chicken embryos (chicken embryo origin, CEO) or in a cell culture (tissue culture origin, TCO) are used most often. These vaccines are used both preventively and metaphylactically to control the spread of the virus and shorten the duration of outbreaks. Although vaccination with live vaccines is effective, vaccine viruses can potentially revert to virulence during passage between vaccinated and unvaccinated birds. Therefore, it is common practice for live-attenuated ILT vaccines to be used only in endemic areas [67,70,71]. For example, in pheasants, attenuated vaccines have been described to cause severe disease [62,73]. Therefore, only genetically modified ILT vaccines should be used, as these do not contain the whole virus and, consequently, cannot spread between animals [73]. These vaccines are usually ophthalmicly administered. In animals with latent *Mycoplasma* sp. infection, this can result in temporarily severe eye swelling. To avoid these adverse effects, these vaccines can be used orally as well [74].

Vectormune^®^ FP ILT + AE (Ceva-Phylaxia Co., Ltd., 1107 Budapest, Hungary) confers protection against FP, ILT, and AE, see Section 3.1.1. The leaflet states that the onset of immunity starts at three weeks for the ILT virus after vaccination and lasts 57 weeks.

Innovax^®^-ILT-IBD (Intervet International B.V., 5831 AN Boxmeer, The Netherlands) is used in other Galliformes than chickens, see Section 3.2.2. It is a highly effective method of ILT control and provides safe, long-term protection.

#### 3.2.4. Pigeon, Falcon, and Owl Herpesvirus

Sequence analysis has revealed that falcon (FaHV-1) and owl (SHV-1) herpesviruses are identical to the *Mardivirus columbidalpha 1* (formerly *Columbid herpesvirus 1)* [75]. *Mardivirus columbidalpha 1* is described to be a highly lethal disease in hawks, falcons, and owls. The clinical signs of herpesvirus infections in falcons and owls, also termed ‘inclusion body disease’ or ‘herpesvirus hepatitis’, consist of general depression and anorexia before death, or sudden death without preceding clinical signs [41,76,77,78,79,80,81,82].

Pharmavac PHA^®^ (Pharmagal-Bio spol. s.r.o, 949 01 Nitra, Slovak Republic), a 3-in-1 vaccine, contains inactivated pigeon paramyxovirus type 1 (PPMV1) (strain 988M), *Mardivirus columbidalpha 1* (strain V298/70), and fowl adenovirus type 8. It can be used for active immunization of pigeons from the age of four weeks onwards. The vaccine is used to reduce morbidity and mortality associated with PPMV1 and adenovirus types 7/E, 2/D, 3/D, and 4C, and to reduce morbidity, mortality, and viral shedding associated with *Mardivirus columbidalpha 1*. The vaccine should be administered SC in the dorsal region of the neck, towards the tail. Immunity takes up to three weeks to develop after vaccination and lasts for at least 12 months after vaccination for PPMV1 and five months for *Mardivirus columbidalpha 1* and adenovirus.

Pharmavac Columbi 2^®^ (Pharmagal-Bio spol. s.r.o, 949 01 Nitra, Slovak Republic) contains inactivated PPMV1 (strain La Sota) and *Mardivirus columbidalpha 1* (strain V298/70). The vaccine is licensed for pigeons for SC and IM administration, and the vaccine can be administered to animals from five weeks of age. The leaflet states that a booster vaccination should be performed after three to four weeks. Adult pigeons should receive a booster yearly, or twice a year in poor epizootiological conditions. In poultry, the onset of immunity is 10–14 days after the first injection, and immunity lasts for one year.

A vaccine trial with an experimental attenuated falcon herpesvirus vaccine was successful in Gyr hybrids (*n* = 3) (*Falco rusticolus* × *F. cherrug* and *F. rusticolus* × *F. biarmicus*) [83]. The vaccine (DuFaHe, Dubai Falcon Herpes) was administered twice SC with a two-week interval. Eighteen days after the booster, falcons were challenged intranasally and ophthalmicly with 0.5 mL of a virulent American falcon herpesvirus isolate. All vaccinated falcons produced serum-neutralizing antibodies after vaccination, and the vaccine conferred complete protection against clinical signs upon virus challenge [83].

Additionally, an autogenous vaccine was developed and used successfully in domestic pigeons, anecdotally decreasing herpesvirus morbidity. However, no formal safety or effectiveness studies have been conducted for this vaccine (Leus, pers. comm).

#### 3.2.5. Duck Viral Enteritis (Duck Plague)

Natural susceptibility to duck viral enteritis (DVE) has been limited to members of the family *Anatidae* (ducks, geese, and swans) of the order Anseriformes [84]. The morbidity and mortality of this virus are described to be very high in adult Muscovy ducks (*Cairina moschata*) [42,84,85,86]. The clinical signs include lethargy, diarrhea, dehydration, and acute death. Since vaccines are presumed to be effective only in DVE naïve birds, and viral shedding is not yet completely understood, vaccination alone cannot be relied upon entirely [84,87].

Kapevac^®^ (Ceva-Phylaxia Co., Ltd., 1107 Budapest, Hungary) and Vaxiduk^®^ (Boehringer Ingelheim Animal Health France SCS, 69007 Lyon, France) can be used metaphylactically due to their rapid onset of immunity. They are not licensed for use in wild ducks. Kapevec^®^ is administered SC under the skin of the neck, and Vaxiduk^®^ is administered SC or IM. For both vaccines, an initial booster should be administered two–three weeks after primary vaccination, with subsequent annual revaccination.

### 3.3. Avian Influenza

Avian influenza (AI) is caused by influenza A viruses and is typically subdivided into two groups based on their pathogenicity: High pathogenic avian influenza (HPAI) and low pathogenic avian influenza (LPAI). The HPAI is restricted to subtypes H5 and H7, although not all H5 and H7 viruses cause HPAI. All other subtypes, and some H5 and H7, cause a milder disease and are thus considered LPAI. It is important to state that AI is considered a zoonosis with significant public health implications, and vaccinations are frequently subject to stringent local regulations [88].

Experimental vaccination of zoo birds against HPAI has mostly been performed with inactivated whole virus vaccines. Results have been mixed, with variable immune responses reported among species [13,89,90,91,92,93,94,95,96]. Serological differentiation of infected and vaccinated animals (DIVA) is possible using a specific neuraminidase (N)-antibody test, but only if the vaccine contains an N-type antigen heterologous to the circulating field virus. Interpretation of post-vaccination serological surveillance in non-domesticated avian species remains challenging, as vaccine-induced antibodies may be indistinguishable from those arising from natural infection. In the absence of validated DIVA strategies for many non-domesticated species, serological data alone may therefore provide limited insight into population-level protection [9,97].

As of May 2024, only two veterinary vaccines are authorized by the European Medicines Agency in the European Union (EU) for the prevention of avian influenza A, subtype H5, in chickens.

Nobilis^®^ Influenza H5N2 (Intervet International B.V., 5831 AN Boxmeer, The Netherlands) is an inactivated avian influenza virus Type A subtype H5N2, suitable for vaccination against all H5 subtypes. The vaccine is marketed for IM or SC administration. The manufacturer’s data sheet states that the primary vaccination, administered to all poultry irrespective of age, can be followed by a booster vaccination four to six weeks later. If primary vaccination is administered before three weeks of age, booster vaccination is recommended at 16 to 18 weeks of age. Under all circumstances, vaccination schedules implemented should comply with the requirements of national regulatory authorities.

Innovax^®^ ND-H5 (Intervet International B.V., 5831 AN Boxmeer, The Netherlands) is licensed for SC administration to reduce mortality, clinical signs, and virus excretion due to infection with HPAI virus of the H5 type. The leaflet states that the onset of immunity is two weeks, but the duration of immunity lasts only 12 weeks. The live vaccine strain is excreted by vaccinated birds and may spread to turkeys. Safety trials have demonstrated that the vaccine strain is safe for turkeys. Nevertheless, precautionary measures should be taken to prevent spillover, as the pathogenicity of the vaccine strain in other non-domesticated bird species remains unknown.

Experimental recombinant vaccines, synthesized in plant leaf tissue, insect, or bacterial cells, offer alternatives to conventional inactivated vaccines. Many doses may be rapidly synthesized once a field virus has been genetically sequenced, with no live virus required. The absence of internal viral proteins, such as nucleoprotein NP, allows for DIVA using standard ELISA tests. In one study, African penguins (*Spheniscus demersus*) were vaccinated with either a conventional inactivated clade 2.3.4.4b H5N8 HPAI whole virus or a tobacco leaf-produced H5-based virus-like particle (VLP) [98]. A single dose of the inactivated vaccine induced protective anti-H5N8 antibody titers; however, antibody levels declined by day 56 and did not reach thresholds considered sufficient to reduce viral shedding. Administration of a booster vaccination resulted in antibody titers exceeding the level required to suppress viral shedding. Nevertheless, no challenge study was conducted to confirm the induction of protective immunity. Although the VLP vaccine requires individual injection of birds, the absence of a booster requirement to confer protection against clinical disease, compared with inactivated vaccines, represents a potential advantage. However, the protective efficacy of this vaccine in vulnerable species should be further validated, and more practical application methods should be explored. In particular, the development of mucosally administered droplet or spray vaccines may help overcome the logistical constraints associated with mass parenteral vaccination.

### 3.4. Avian Paramyxovirus 1

Avian paramyxoviruses (APMV) belong to the family *Paramyxoviridae*, genus *Avulavirus*. Newcastle disease (ND) virus (*Orthoavulavirus javaense*; APMV-1) is the most well-recognized member of the 12 APMV serotypes [99,100]. ND is a highly contagious and often fatal viral disease that has been reported in a wide variety of avian species worldwide, including African penguins, little owls (*Athene noctua*), bearded vultures (*Gypaetus barbatus*), Eurasian scops owls (*Otus scops*), ostriches, southern ground-hornbill (*Bucorvus leadbeateri*), cockatiels (*Nymphicus hollandicus*), and gray parrots (*Psittacus erithacus*) [101,102,103,104,105,106,107,108,109,110].

APMV-1 strains originating from Columbiformes are antigenically and genetically distinct from poultry-derived strains, and therefore, they are called pigeon paramyxovirus type-1 (PPMV-1) [111]. The described clinical signs are a sudden onset of anorexia, respiratory signs (i.e., dyspnea), diarrhea, paralysis, and acute death in adults, resulting in a high mortality rate [6,112]. ND has zoonotic potential [110,113].

Pestikal^®^ La Sota SPF (Genera, d.d., 10436 Kalinovica, Croatia) was administered in adult ostriches, hybrids between blue-neck and African black ostriches (*n* = 24), to study the best administration route in a manner adapted to the ostrich anatomy. They compared the effect of oral administration in drinking water, spraying, and the oculo-nasal administration route on specific antibody titers. In ostriches, it was observed that antibody production with titers sufficient for humoral immunity in all experimental groups occurred after vaccination with Pestikal^®^ La Sota SPF. Regardless of the administration route, no adverse reactions to the vaccine had been observed. In comparison to the vaccine administration via drinking water, oculo-nasal administration and spraying induced a better humoral response manifested by higher specific antibody titers [102].

Avishield^®^ ND B1 (Genera d.d, 10436 Kalinovica, Croatia) is a live-attenuated vaccine containing a lentogenic ND Hitchner B1 strain. It is licensed for use in broilers and layers in the rearing phase. Immunity is obtained three weeks after vaccination and lasts for five weeks post vaccination. The vaccine has been used in ring-necked pheasants, with no adverse effects observed (Leus, pers. comm). Data on vaccine effectiveness in non-domesticated species remain absent.

Nobilis^®^ RT + IBmulti + ND + EDS (Intervet International B.V., 5831 AN Boxmeer, The Netherlands) is a combined inactivated vaccine for active immunization against avian pneumovirus, infectious bronchitis virus (IBV), ND, and Egg Drop Syndrome (EDS). Anecdotally, the vaccine has been administered without observed adverse effects to Indian peafowl, Japanese quail, gray peacock-pheasants (*Polyplectron bicalcaratum*), Temminck’s tragopans (*Tragopan temminckii*), green pheasants (*P. versicolor*), ring-necked pheasants, Edwards’s pheasants (*Lophura edwardsi*), and silver pheasants (*L. nycthemera*). The efficacy for NCD, however, was not tested by challenge, since it was administered in a field setting (Leus, pers. comm).

Innovax^®^-ND-IBD (Intervet International B.V., 5831 AN Boxmeer, The Netherlands) is a live HVT vector vaccine licensed for use in poultry that confers protection against IBD, ND, and MD. The vaccine has been administered to domestic ducks, turkeys, ring-necked pheasants, Indian peafowl, and Japanese quail with no adverse effects (Leus, pers. comm). However, no claims can be made about vaccine effectiveness in these species.

Nobilis Ma5 + Clone 30 (MSD Animal Health, 85716 Unterschleissheim, Germany) is registered for the immunization of fowl against ND. The vaccine is administered by (coarse) spray, oculonasal administration, or in the drinking water. The leaflet states that adequate immunity against ND will last for approximately six weeks. In areas where ND is endemic, a booster should be given. The vaccine has been administered orally to capercaillie (*Tetrao urogallus*), black grouse (*Lyrurus tetrix*), and rock ptarmigan (*Lagopus muta*) without observed adverse effects (Kempf, pers. comm).

One study described the use of three ND vaccines (two live and one killed) tested in houbara bustards. All resulted in immunity exceeding nine months of duration, with the inactive vaccine in adjuvant consistently resulting in the highest titers after two SC vaccinations four weeks apart [114].

In falcons (*Falco* spp.), the use of a killed vaccine is described [29,30,31]. The vaccine contained strains from four different avian species and was produced by the Central Veterinary Research Laboratory in Dubai (UAE). Prime vaccination should be performed at the age of three to nine weeks, and a booster vaccination four weeks later. Since the introduction of this vaccine, the morbidity of ND in their falcons has decreased drastically [29,30,31].

Various vaccination schedules and administration routes (ophthalmic, SC, IM, oral) were studied in southern ground-hornbill (*n* = 75) [104]. Four ND virus strains or vaccines from commercially available products in South Africa were used. Vaccination with a live ND strain ophthalmicly administered, followed by SC administration of the Struvac vaccine (Deltamune, 0002 Pretoria, South Africa), elicited antibody levels considered sufficient to protect the species against natural ND infection. However, no clinical challenge trials were conducted in this study.

In poultry, attenuated and inactivated ND vaccines are combined to provide improved and long-acting protection. In zoological settings, similar combinations have been studied with various bird species, with some examples described below.

Live-attenuated ND strains Hitchner B1 and La Sota, as well as an inactivated ND vaccine, were administered to ring-neck pheasants, cockatiels, rainbow lorikeets (*Trichoglossus haematodus molucanos*), eastern rosellas (*Platycercus eximius*), Guinea turacos (*Tauraco persa*), white-cheeked turacos (*T. leucotis*), and violet turacos (*Musophaga violacea*). With the exception of psittacines, the birds exhibited a marked increase in antibody titers [115].

In bustard chicks (*Otididae*), a live Hitchner B1 strain vaccine may be administered via the ophthalmic or intranasal route at 4, 8, and 20 weeks of age. A second vaccine (Nobilis^®^ Newcavac; Intervet International B.V., 5831 AN Boxmeer, The Netherlands) should be administered at 32 weeks of age, followed by annual revaccination with Nobilis^®^ Newcavac [116].

For PPMV-1 in pigeons, several vaccines are commercially available in Europe, including Pharmavac PHA^®^ (see Section 3.2.4), Pharmavac columbi 2^®^ (see Section 3.2.4), RP Vacc^®^ (Pharmagal-Bio spol. s r.o, 949 01 Nitra, Slovak Republic), Nobilis^®^ Paramyxo P201 (Intervet International B.V., 5831 AN Boxmeer, The Netherlands), and Colombovac^®^ PMV (Zoetis Belgium SA, 1930 Zaventem, Belgium).

RP Vacc^®^ is an inactivated vaccine against PPMV-1 and pigeon rotavirus (PiRV). The vaccine should be administered SC or IM. According to the manufacturer’s recommendations, primary vaccination of young pigeons is performed at four weeks of age, followed by a second dose three weeks later. Booster vaccinations are administered annually; however, in areas with high infection pressure, the interval may be reduced to 8–9 months. Protective immunity is achieved approximately 14 days after primary vaccination and persists for approximately nine months against PPMV-1 and eight months against PiRV.

Nobilis^®^ Paramyxo P201 is an inactivated vaccine against PPMV-1 and contains a PPMV-1 antigenic P201 strain. The vaccine should be administered SC at a minimum age of five weeks. The onset of immunity occurs approximately four weeks after administration and persists for one year. Subsequent booster vaccinations are recommended annually.

Colombovac^®^ PMV is an inactivated vaccine against PPMV-1 and, in contrast to the vaccines described above, contains the LaSota strain of PPMV-1. The vaccine may be administered subcutaneously to pigeons from three weeks of age. Protective immunity is achieved approximately one month after vaccination and lasts for one year, after which annual booster vaccinations are recommended.

Caution should be exercised when using live ND vaccines in avian species, considering the potential risk of introducing a virus or adding viral genetic variation into naïve, non-target populations. However, considering that captive bird species are expected to remain in captivity and that biosecurity of premises is of interest for owners, the spillover risk to the native avian fauna may be limited, especially in well-designed aviaries. Vaccination of high-risk and high-value birds in managed collections may therefore be considered to reduce severe disease and mortality. However, such vaccination strategies may not fully prevent infection or viral shedding [99,117,118,119,120]. Future research should focus on determining vaccine protective efficacy in non-domesticated avian species.

### 3.5. Parrot Bornaviruses (PaBV)

Parrot bornaviruses (PaBV), or avian bornaviruses, are a group of *Orthobornavirus* species that may cause avian ganglioneuritis, a peracute to chronic and often fatal condition typically associated with a range of neurological and/or gastrointestinal symptoms [121]. PaBV disease is suggested to be immune-mediated and to involve a complex pathogenesis. Avian bornavirus (ABV) infections have been described in a wide range of families within the order Psittaciformes, and it is assumed that the vast majority of psittacine species are susceptible to infection, as the virus is observed in captive psittacine populations worldwide [122].

At present, neither effective therapies nor immunoprophylaxis are available for PaBV infection or avian ganglioneuritis, despite their substantial impact on private psittacine collections and conservation breeding programs [122,123]. Because ABV-associated disease is primarily T-cell mediated, vaccination could theoretically exacerbate disease. Nevertheless, several studies have evaluated the protective efficacy of experimental vaccines in psittacines (cockatiels) and non-psittacine birds (canaries) [124,125,126,127]. Outcomes ranged from no measurable protection to birds remaining largely free of clinical disease despite persistent viral presence in multiple organs. Collectively, these findings suggest that vaccination may protect against clinical disease without exacerbating infection, although it does not eliminate viral persistence.

### 3.6. Avian Polyomaviruses

Avian polyomaviruses (APV) belong to the genus *Gammapolyomavirus*. APV has a broad host range and is known to cause acute to chronic disease in a wide range of psittacine species, including parrots, cockatoos, macaws, and budgerigars [46,128]. Many cases of APV-associated disease have been reported in young captive birds and are often characterized by high mortality rates. Clinical manifestations may include hepatitis, ascites, pericardial effusion, and abdominal distension. Adult and chronically infected parrots often exhibit abnormal feather growth [129].

The use of experimental inactivated virus vaccines to control APV outbreaks in psittacine birds has been described [130,131,132,133]. Although vaccination has been shown to be safe, immunogenic, and protective under controlled experimental conditions in certain psittacine species, evidence demonstrating consistent clinical protection across diverse non-domesticated avian taxa remains limited. Consequently, antibody detection alone should be interpreted cautiously when extrapolating vaccine efficacy beyond the species and experimental conditions studied.

According to product information, the commercial Psittimune^®^ Avian Polyomavirus Vaccine (Psittimune^®^ APV; Creative Science LLC, Ballwin, MO, USA) is reported to reduce mortality associated with APV infection in psittacines. However, we were unable to identify peer-reviewed studies that substantiate this claim. In addition, this vaccine does not appear to be readily available in Europe.

### 3.7. Circovirus parrot

*Circovirus parrot*, formerly known as beak and feather disease virus, is the causative agent of psittacine beak and feather disease (PBFD). The disease primarily affects parrots and is characterized by progressive feather dystrophy, beak deformities, anorexia, diarrhea, lethargy, dehydration, immunosuppression, and high mortality rates, particularly in juvenile birds [46,134,135,136,137]. Although PBFD was initially documented in psittacines in Australia, the virus has now attained global distribution [137,138].

Although no commercial PBFD vaccines are available, experimental vaccines have been tested in two studies with promising results [139,140]. In study one, seven wild-caught galah (*Eolophus roseicapillus*) nestlings and 15 sulphur-crested cockatoos (*Cacatua galerita*) were vaccinated and subsequently challenged with *Circovirus parrot* [139]. The purpose was to compare an inactivated *Circovirus parrot* vaccination regimen with a double oil emulsion adjuvant system. Both vaccine formulations protected chicks from experimental challenge.

In the second study, long-billed corellas (*Cacatua tenuirostris*) (*n* = 30) were vaccinated IM on days 0 and 11 with a recombinant PBFD virus capsid protein vaccine [140]. Subsequently, vaccinated birds and non-vaccinated control birds (*n* = 5) were challenged IM and orally 16 days after the second vaccination. Vaccinated birds did not develop feather lesions, had only transient PCR-detectable viraemia, and had no evidence of persistent infection 270 days post-challenge [140].

Experimental vaccination may induce protective antibodies that can be transferred to offspring. However, circoviruses cannot yet be cultured in vitro, posing a major obstacle to the development of vaccines [141]. Consequently, future research may focus on the development of a recombinant vaccine.

### 3.8. West Nile Virus (WNV)

*West Nile Virus* (*Orthoflavivirus nilense*; WNV) is maintained in nature through transmission cycles between mosquitoes and birds and may also infect humans and horses worldwide [142,143]. Clinical disease is most commonly characterized by neurological manifestations, including encephalitis or meningoencephalitis [6].

Several studies have addressed the protective efficacy of commercial and experimental WNV vaccines using different platforms, administration routes, and vaccination regimens. Study outcomes varied greatly in domestic and wild avian species [12,144,145,146,147]. Direct comparison among studies is challenging due to substantial heterogeneity with respect to the vaccine type used, vaccination schedules, administration routes, doses, sample size, viral strains, methodologies, and analyzed variables.

Cross-protection between WNV and emerging flaviviruses in wild birds has been suggested [148]. Indeed, prior infection with related flaviviruses, such as Usutu virus, has been shown to protect domestic greylag geese (*Anser anser*) and magpies (*Pica pica*) from severe WNV disease [149,150].

Vaccines originally developed for horses, i.e., West Nile-Innovator^®^ (Zoetis Belgium SA, 1348 Louvain-la-Neuve, Belgium) and PROTEC^®^ Equine *West Nile Virus* (Boehringer Ingelheim, 69007 Lyon, France), appear to be the most used commercial vaccines in avian species, such as cranes, raptors, penguins, flamingos, and corvids [12,146,151,152]. However, their use in birds should be approached with caution due to the lack of controlled safety and efficacy trials in non-domesticated avian species.

Limited efficacy of vaccination in captive-bred mature gyrfalcons (*Falco rusticolus*) and hybrid falcons (*F. rusticolus* × *F. cherrug* and *F. rusticolus* × *F. peregrinus*) has been demonstrated [153]. Two commercial vaccines were tested, i.e., an inactivated vaccine, Duvaxyn^®^ WNV, Fort Dodge Animal Health, Overland Park, KS, USA (in the EU now commercial available as Equip^®^ WNV, Zoetis Belgium S.A., 1930 Zaventum, Belgium; in the USA commercial available as West Nile-Innovator^®^ + VEWT, Zoetis Inc., Parsippany, NJ, USA), and a recombinant live vaccine (Recombitek^®^-Equine WNV vaccine (Merial Ltd., Athens, GA, USA), approved in the EU as Proteq West Nile (Boehringer Ingelheim, 69007 Lyon, France)). It has been demonstrated that Recombitek^®^ provides a slightly better protection than Duvaxyn^®^, but moderate (Recombitek^®^) and weak (Duvaxyn^®^) adverse effects were observed at the injection sites. Injection-site lesions included extensive granulomatous and pyogranulomatous myositis with the use of Recombitek^®^, and pustules, induration, and slight swelling with the use of Duvaxyn^®^. For both vaccines, administration of a third vaccine dose resulted in attenuation of clinical signs, reduced viraemia, and absence of mortality, although injection-site adverse effects were not entirely eliminated.

Another study described the use of West Nile-Innovator^®^, an experimental DNA plasmid vaccine, and Recombitek^®^ in California scrub jays (*Aphelocoma californica*) [154]. None of the tested vaccines induced sterilizing immunity following a single vaccination, and none were entirely free of adverse effects. The West Nile-Innovator provided the best protective immune priming with the least side effects. However, the small sample sizes (*n* = 6 per group) limit the statistical strength of these findings. Nevertheless, considering the well-documented high morbidity and mortality associated with WNV infection in corvids, even a partially effective vaccine may be beneficial in vulnerable populations.

An experimental DNA vaccine has been safely used in captive Andean (*Vultur gryphus*) and California (*Gymnogyps californianus*) condors [155]. Two IM vaccinations administered three weeks apart induced protective neutralizing antibody titers in adults, nestlings, and newly hatched chicks and protected vaccinated captive birds from naturally circulating WNV.

### 3.9. Eastern and Western Equine Encephalitis

Eastern equine encephalitis virus (*Alphavirus eastern*; EEEV) and western equine encephalitis virus (*Alphavirus western*; WEEV) are important zoonotic, vector-borne alphaviruses that are maintained in a cycle involving birds and mosquitoes [156,157]. Although birds serve as the primary amplifying hosts, EEEV can cause clinical disease in several avian species, including emus (*Dromaius novaehollandiae*), southern cassowaries (*Casuarius casuarius*), sandhill cranes (*Grus canadensis*), whooping cranes (*G. americana*), psittacines, great egrets (*Ardea alba*), turkeys, and ostriches [6,158,159,160,161,162,163]. WEE has been diagnosed in emus and different passerine species, e.g., purple finches (*Haemorhous purpureus purpureus*), northern cardinals (*Cardinalis cardinalis*), house sparrows (*Passer domesticus*), white-throated sparrows (*Zonotriohia albicollis*), towhees (*Pipilo erythrophthalmus*), and blue jays (*Cyanocitta cristata*) [164,165]. Clinical signs in affected birds include incoordination, paresis, and progressive paralysis; feather picking and cannibalism have also been observed, particularly in densely housed populations [6].

A vaccine trial was conducted in emus (*n* = 25) aged 12–14 months to evaluate four vaccine formulations: two commercially available polyvalent vaccines licensed for equine immunization, Equiloid (Fort Dodge Animal Health, Overland Park, KS, USA) and Triple-E (Solvay Animal Health, Mendota Heights, MN, USA), and two experimental monovalent vaccines developed for human use [158]. All four vaccines elicited measurable neutralizing antibody responses. Following vaccination, the birds were challenged with a normally lethal dose of EEEV. Complete protection was observed in all vaccinated emus, whereas the unvaccinated control group developed viraemia. The European equivalents are Equiloid Innovator^®^ (Zoetis Belgium S.A., 1930 Zaventem, Belgium) and Core EQ Innovator^®^ (Zoetis Belgium S.A., 1930 Zaventem, Belgium). In horses, these vaccines are administered IM with an annual booster vaccination in the spring. However, no safety or efficacy studies have been conducted for these vaccines in avian species.

### 3.10. Reovirus

Avian reoviruses (ARV) are transmitted primarily via the fecal–oral route. While not all viral strains are associated with clinical disease, the most-observed condition in poultry is viral tenosynovitis, which may progress to arthritis. Additional clinical signs vary depending on viral strain and host susceptibility, and may include reduced growth rates, poor feed conversion, depression, ruffled feathers, diarrhea, and, in severe cases, increased mortality. Subclinical infections are common and may contribute to immunosuppression and increased susceptibility to secondary infections [166,167].

The use of Nobilis^®^ Reo Inac (Intervet International B.V., 5861 AN Boxmeer, The Netherlands) in bustards is anecdotally described [33]. This inactivated ARV-1 vaccine is licensed for IM or SC administration in chickens. According to the manufacturer’s instructions, the best protection against ARV is achieved when chickens receive a primary vaccination with a live ARV-1 vaccine, e.g., Nobilis^®^ Reo 1133 (Intervet International B.V., 5831 AN Boxmeer, The Netherlands) or Nobilis^®^ Reo 2177 (Intervet International B.V., 5831 AN Boxmeer, The Netherlands), six weeks before vaccinating with Nobilis^®^ Reo Inac.

Nobilis^®^ Multriva RT + IBm + ND + Gm + REOm + EDS (Intervet International B.V., 5831 AN Boxmeer, The Netherlands) is a more recent inactivated vaccine licensed for IM use in animals from eight weeks of age. This vaccine contains both inactivated ARV-1 and ARV-4. Similar to the Nobilis^®^ Reo Inac, the vaccine is intended to be used as a booster after initial vaccination with the live Nobilis^®^ Reo 1133 or Nobilis^®^ Multriva REOm (Intervet International B.V., 5831 AN Boxmeer, The Netherlands). While this vaccine has not been assessed in non-chicken species, it merits mention due to the inclusion of multiple ARV genotypes (ARV-1 and ARV-4) in combination with antigens targeting other clinically important diseases of galliform species, including avian metapneumovirus (RT), IBD, ND, avian infectious bursal disease (Gumboro disease; Gm), and EDS (Leus, pers. comm).

In chickens, studies evaluated the safety and efficacy of autogenous vaccines against ARV [168]. In broiler breeder flocks, these autogenous ARV vaccines are frequently employed to confer protection to their offspring through maternally derived antibodies. This strategy may also represent a promising approach for other avian species when a locally circulating strain is associated with disease (Leus, pers. comm).

### 3.11. Adenovirus

#### 3.11.1. Marble Spleen Disease

Marble spleen disease (MSD) is an infectious disease of pheasants and guineafowl (*Numida meleagris*) caused by a siadenovirus, which is the same virus that causes hemorrhagic enteritis in turkeys [169,170]. Characteristic lesions include splenic discoloration and/or enlargement, as well as acute multi-organ congestion. The disease is peracute, with young adult birds often dying following a very short clinical course. Birds aged four to eight months are particularly susceptible [6]. In pheasants, disease prevention has been achieved through administration of an attenuated turkey hemorrhagic enteritis vaccine. However, caution is warranted, as some vaccine strains originate from pheasants and may retain residual pathogenicity. Therefore, the use of the vaccine in guineafowl is not recommended [171,172].

Dindoral^®^ SPF (Boehringer Ingelheim Vetmedica GmbH, 55216 Ingelheim am Rhein, Germany) is a commercially available freeze-dried vaccine for administration via drinking water for turkeys. It can be administered to poultry from the age of 28 days. The leaflet described that the onset of immunity is one week after the first administration and lasts for four months.

#### 3.11.2. Pigeon Adenovirus

There are two adenovirus-associated syndromes that have been described in domestic pigeons. Pigeon adenovirus type I (*Aviadenovirus columbae*; PiAdV-1) primarily affects young birds and is associated with diarrhea, vomiting, weight loss, and high morbidity and mortality. Pigeon adenovirus type 2 (*Aviadenovirus columbidae*; PiAdV-2) causes necrotizing hepatitis [173,174] in wild Eurasian collared doves (*Streptopelia decaocto*) and Eurasian spotted doves (*S. chinensis*). PiAdV genotypes 4 and 5 have been detected by PCR but are considered nonpathogenic [175].

Within the EU, a commercial vaccine is available (Pharmavac PHA^®^, see Section 3.2.4).

### 3.12. Infectious Bronchitis Virus (IBV)

Infectious bronchitis virus (*Gammacoronavirus galli*; IBV) is an avian coronavirus that primarily causes respiratory, renal, and female reproductive tract disease in chickens. Although chickens and pheasants are reported to be natural hosts of IBV, clinically apparent IBV infections are predominantly reported in chickens [176,177,178,179,180,181]. IBV may cause enteric disease in turkeys [182].

Commercial live-attenuated vaccines licensed for poultry are available to reduce respiratory disease associated with IBV. Poulvac^®^ IB QX (Zoetis Belgium SA, 1930 Zaventem, Belgium) is administered by spray vaccination, with onset of immunity approximately three weeks after vaccination and a duration of nine weeks. Poulvac^®^ IB Primer (Zoetis B.V., 2909 LD Capelle aan den IJssel, The Netherlands) may be administered by spray, ophthalmic, or drinking water. Onset of immunity occurs approximately 27 days after vaccination, and the duration of immunity is 26 weeks. However, vaccine virus shedding may result in transmission to in-contact birds for at least 14 days following vaccination.

An IBV QX-like strain was diagnosed by PCR in a flock of ring-necked pheasants during maturation. The animals were vaccinated with Poulvac^®^ IB QX and Poulvac^®^ IB Primer. Morbidity decreased, and no adverse effects were observed (Leus, pers. comm).

Nobilis^®^ RT + IBmulti + ND + EDS (strains IBV 249 and IBV M41) has been used off-label to different Galliformes (see Section 3.4) (Leus, pers. comm).

Nobilis Ma5 + Clone 30 (MSD Animal Health, Unterschleissheim, Germany) was orally administered off-label to capercaillie, black grouse, and rock ptarmigan (see Section 3.4) (Kempf, pers. comm).

### 3.13. Avian Metapneumovirus

Avian metapneumovirus (*Metapneumovirus avis*; aMPV) is the etiological agent responsible for swollen head syndrome (SHS) in chickens and turkey rhinotracheitis (TRT) in turkeys. Although aMPV is most associated with chickens and turkeys, it may also infect ducks, pheasants, and guinea fowl [183,184,185,186,187]. Most aMPV infections are subclinical or asymptomatic. When clinical disease occurs, it is typically characterized by mild respiratory signs and reproductive disturbances. Severe disease is uncommon but may include pronounced respiratory symptoms and, in some cases, neurological abnormalities [188]. Despite this, the role of aMPV as a causative or predisposing agent in the avian respiratory disease complex is often underestimated. Secondary infections and colonization by opportunistic bacteria can exacerbate clinical disease and contribute to increased tissue damage. Although host adaptation appears to be of limited concern, the high mutation rate of aMPV may result in the emergence of genetically and phenotypically distinct clusters, potentially affecting vaccine effectiveness [189]. It is suggested that human metapneumovirus has evolved from aMPV following zoonotic transfer [190].

Several studies have evaluated the protective immunity induced by inactivated aMPV subtype B vaccines in chickens, demonstrating sustained protection for up to six months post-vaccination [191,192,193].

Ring-necked pheasants (*n* = 18,120) have been vaccinated with the live-attenuated Nobilis^®^ TRT vaccine (Intervet Nederland B.V., 5831 AN Boxmeer, The Netherlands) at one day and five weeks of age. Seroconversion was confirmed in blood samples obtained three weeks post-vaccination. In 33% of the population, positive titers were maintained up to at least 22 weeks of age [194].

Nobilis^®^ RT + IBmulti + ND + EDS has been safely administered to various Galliformes species (see subheader 3.4) (Leus, pers. comm). However, the extent of protection it confers against aMPV remains unknown.

### 3.14. Goose Parvovirus

*Dependoparvovirus anseriform 1*, commonly referred to as goose parvovirus, is the causative agent of Derzsy’s disease in young geese and Muscovy ducks and may result in high mortality in goslings [195]. This disease has also been reported in Canada geese (*Branta canadensis*) and greater snow geese (*Anser caerulescens atlanticus*) [196]. In Germany, a serological survey in bean geese (*A. fabalis*) and white-fronted geese (*A. albifrons*) demonstrated that 48% of wild animals had neutralizing antibodies [197].

Vaccination of geese and Muscovy ducks is possible using Deparmune^®^ (Ceva Santé Animale BV, 2671 SB Naaldwijk, The Netherlands). This inactivated vaccine may be administered SC or IM at one day of age, followed by a booster vaccination three weeks later.

## 4. Bacterial Etiology

### 4.1. Avian Pathogenic Escherichia coli (APEC)

Avian pathogenic *Escherichia coli* (APEC) causes colibacillosis in poultry, including chickens, turkeys, ducks, and many other avian species, and has been suggested as a potential foodborne zoonotic pathogen. Clinical signs and pathological findings include perihepatitis, airsacculitis, pericarditis, egg peritonitis, salphingitis, coligranuloma, omphalitis, cellulitis, osteomyelitis/arthritis, and mortality [198,199].

Poulvac^®^ *E. coli* (Zoetis Belgium S.A., 1930 Zaventem, Belgium) is a live-attenuated vaccine containing *E. coli* serotype O78 that can be sprayed onto the animals or administered via drinking water. It is licensed for both turkeys and chickens. The vaccine may be administered from the age of one day by aerosols or five days via drinking water.

Nobilis^®^ *E. coli* inac (Intervet International B.V., 5831 AN Boxmeer, The Netherlands) is recommended to be administered SC or IM. Vaccination may be performed in birds older than six weeks of age, with a booster administered between 14 and 18 weeks of age. The interval between the two doses must be at least six weeks.

Autogenous *E. coli* vaccines, typically formulated as oil emulsions, are widely and effectively used in commercial breeder and layer chickens [200,201,202,203]. However, their use in non-domesticated birds should be approached with caution due to the lack of controlled safety and efficacy studies.

### 4.2. Avian Mycoplasmosis

**Figure 5:**
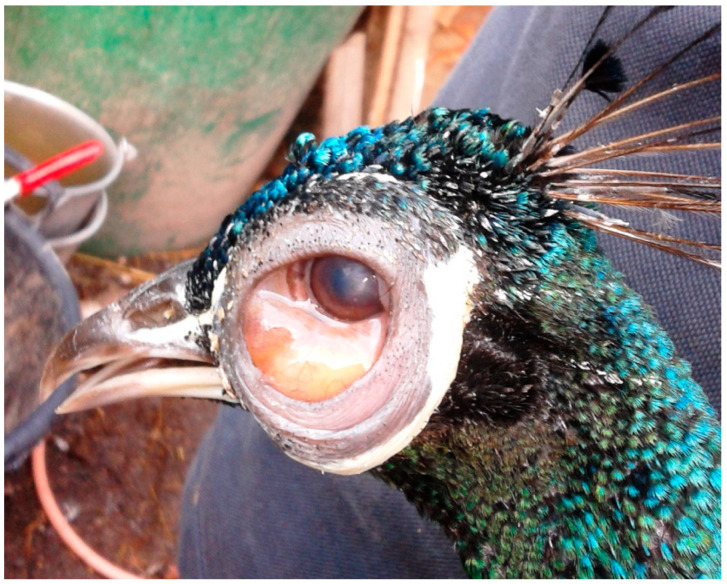
Mycoplasma infection in an Indian peafowl (*Pavos cristatus*) with fibrin debris at the infraorbital sinus (photograph provided by H. Kempf).

*Mycoplasma* spp. are well-known in the poultry industry, but may also cause chronic respiratory disease in non-domesticated bird species [204]. Out of the four *Mycoplasma* spp., *Mycoplasma gallisepticum* (MG) is considered the major pathogen of gallinaceous and certain non-gallinaceous avian species. Clinical symptoms of MG infection include rales, coughing, sneezing, nasal discharge, swollen infraorbital sinuses, and mortality (Figure 5). MG is described in house finches (*Haemorhous mexicanus*), American goldfinches (*Spinus tristis*), purple finches (*H. purpureus*), house sparrows, pheasants, chukar partridges (*Alectoris chukar*), gray partridges (*Perdix perdix*), ring-necked pheasants, peafowl, Japanese quail, bobwhite quail, domestic pigeons, ducks, geese, yellow-naped Amazon parrots (*Amazona ochrocephala*), greater flamingos (*Phoenicopterus roseus*), and peregrine falcons (*F. peregrinus*) [201,202,203,204,205,206,207,208,209]. *Mycoplasma synoviae* (MS) has been described in guineafowl and red-legged partridges (*Alectoris rufa*) [210,211].

Limited efficacy has been claimed for the off-label use of MG vaccines under the cascade approach to prevent clinical mycoplasmosis; however, no supporting data were provided [212].

A commercial live MG vaccine, FVAX-MG^®^ (Intervet South Africa (Pty) Ltd., 1619 Spartan, South Africa), containing the F strain of MG, is available for administration by spray application. According to the manufacturer’s leaflet, FVAX-MG^®^ should not be administered within one week before or after vaccination with live ND, IBV, or ILT vaccines. In addition, the manufacturer warns that the MG F strain may cause clinical disease in turkeys; therefore, turkeys should not be exposed to recently vaccinated birds. Given uncertainties regarding efficacy and the potential for spread to non-target species, the use of live MG vaccines should be approached with caution [209].

Nobilis MG 6/85^®^ (Intervet Nederland B.V., 5831 AN Boxmeer, The Netherlands) is considered a safer alternative to the F-strain vaccine and is administered via fine-spray nebulization [213]. The 6/85 strain is regarded as apathogenic and is associated with reduced transmission within flocks compared with the F strain [214,215].

In addition to live vaccines, several inactivated MG vaccines are licensed for use in poultry, including Fixr^®^ MYC-VAC (Kernfarm B.V., 3621 ZB Breukelen, The Netherlands) and Poulvac^®^ MG (Zoetis Belgium S.A., 1930 Zaventem, Belgium). Fixr^®^ MYC-VAC is recommended to be administered twice SC with an eight-week interval. Protective immunity is achieved approximately ten weeks after primary vaccination and is reported to last up to 42 weeks. Poulvac^®^ MG may be administered either SC or IM, with optimal protection achieved following two doses administered at least four weeks apart, followed by an annual booster vaccination. Poulvac^®^ MG has been used in ring-necked pheasants without any reported adverse effects (Leus, pers. comm).

Vaccination against MS may also be considered [216,217,218]. Vaxsafe^®^ MS (Protect A Chick (Pty) Ltd., 0232 Skeerpoort, South Africa) is a live-attenuated vaccine marketed for ophthalmic administration. In poultry, MS-negative birds are recommended to receive a single dose between three and fourteen weeks of age, with vaccination between three and six weeks considered optimal. A single ophthalmic administration confers protection for at least 40 weeks. Vaxsafe^®^ MS is compatible with concurrent administration of other respiratory vaccines, including live MG, IBV, ND, and ILT vaccines. Inactivated vaccines are available as well, for example, Fixr^®^ MS-VAC (Kernfarm B.V., 3621 ZB Breukelen, The Netherlands), which is administered SC in two doses at 10–12 and 18–20 weeks of age. Protective immunity is achieved approximately three weeks after the primary vaccination and reportedly persists for up to 42 weeks.

### 4.3. Erysipelothrix rhusiopathiae

Among the eight *Erysipelothrix* spp. that have been described to date, only *E. rhusiopathiae* appears to be of pathogenic significance to non-domestic bird species, where it is the causative agent of erysipelas [216]. The list of affected species consists of a variety of captive and free-ranging avian species, including turkeys, malleefowl (*Leipoa ocellata*), domestic white Pekin ducks, rainbow lorikeets, eclectus parrots (*Eclectus roratus*), ring-necked pheasants, little blue penguins (*Eudyptula minor*), laughing kookaburras (*Dacelo novaeguineae*), quail, emus, rock partridges (*Alectoris graeca*), king vultures (*Sarcoramphus papa*), kākāpōs (*Strigops habroptilus*), and pied avocets (*Recurvirostra avosetta*) [219,220,221,222,223,224,225,226,227,228,229,230,231,232,233,234]. This bacterium originates from soil, infected animals, and their byproducts. Associated clinical signs include septicemia, pododermatitis, and pericardial hemorrhages, often with high mortality rates [235]. Notably, *E. rhusiopathiae* is a zoonotic pathogen [219].

Under field conditions, emus vaccinated with a commercial vaccine (Vaxall Erysipelas vaccine^®^, Cyanamid-Websters, St. Leonards, Australia) exhibited no clinical signs following experimental challenge with *E. rhusiopathiae* [236]. Emus were vaccinated at four and eight weeks of age with 0.5 mL administered SC. For emus older than three months, the dose was increased to 1 mL for each vaccination. An annual booster was initially recommended for all birds. Although field observations suggest that vaccination under these conditions may confer protection, further studies are required to evaluate vaccine safety and efficacy in other avian species.

A similar commercial vaccine (Commonwealth Serum Laboratories, MelbourneAustralia) was successfully implemented in malleefowl, with no subsequent erysipelas-associated mortality following initiation of the vaccination program. Adult birds were vaccinated SC, with booster vaccinations administered opportunistically because of the risk of injury associated with capturing the birds. No adverse effects were observed following vaccination [227]. In Australia, this vaccine was continued as Eryvac^®^ (Zoetis Australia Pty Ltd., Rhodes, Australia) for the control of Erysipelas-associated arthritis in lambs and sheep.

In kākāpōs, current vaccination recommendations consist of two SC doses of an adjuvanted killed bacterin administered four weeks apart [232,233]. Owing to the species’ relatively short neck and the presence of cervicocephalic air sacs in the cervical region, the neck should be avoided as an injection site. Instead, the vaccine may be administered SC within the inguinal flap.

Pied avocets were vaccinated with Nobilis^®^ Erysipelas (Intervet Nederland B.V., 5831 ABN Boxmeer, The Netherlands), an inactivated vaccine containing the M2 strain of *E. rhusiopathiae*. Birds received a SC injection of 0.3 mL, followed by a booster dose four weeks later, despite the vaccine being licensed for IM administration at a dosage of 0.5 mL. No adverse effects were observed following administration. Although vaccine efficacy has not been formally evaluated, no clinical cases have been recorded since vaccination [234]. This vaccine has been administered at a dose of 0.5 mL per bird in various ibis species for several decades without observing adverse effects or clinical disease (K. Baumgartner, Tiergarten Nürnberg, 90480 Nürnberg, Germany, pers. comm).

The use of orally administered live swine vaccines in commercial cage-free layer chickens and turkeys has shown promising results [237,238]. In layer chickens, primary vaccination was followed by a booster and directly reduced mortality during an ongoing *E. rhusiopathiae* outbreak. However, three months after the booster, mortality returned. According to the manufacturer, vaccine-induced immunity persists for up to four months in swine and appears to be of shorter duration in chickens. In turkeys, vaccination with Hydrovac^®^ (Anchor Laboratories, St. Louis, MO, USA) resulted in incomplete but measurable protection against experimental challenge with *E. rhusiopathiae*, including reductions in mortality and disease severity [238].

### 4.4. Salmonella spp.

Disease due to *Salmonella* spp. infection has been described in species belonging to the orders Passeriformes, Psittaciformes, Galliformes, Anseriformes, and Columbiformes [239,240,241,242,243,244,245,246,247]. In pigeons, one of the most important diseases is paratyphoid, caused by *Salmonella typhimurium* var. *Copenhagen* [248]. Several bird species are considered carriers, including herring gulls (*Larus argentatus*) [249]. These gulls are frequently observed around zoological enclosures and may facilitate interspecies transmission, for example, to penguins [250]. One of the most conspicuous manifestations of salmonellosis is its association with high-mortality “die-offs” in passerine birds, particularly sparrows and finches [251]. *Salmonella* spp. are one of the most important zoonotic pathogenic agents worldwide [251,252,253].

In addition to the risks posed to zoological collections, the impact of *Salmonella* spp. infection in commercial poultry is high. *Salmonella* vaccines are a core component of preventive health management plans in poultry farms [251,254,255,256,257,258].

In Japanese quail, administration of an inactivated *S. gallinarum* (SG) vaccine resulted in increased egg production, reduced mortality, and lower SG loads in the liver and spleen following experimental challenge [259]. In another study, quail were vaccinated with a commercial oil-adjuvanted inactivated vaccine against *S. enteriditis* (SE) and *S. typhimurium* (ST). Although specific details of the vaccine formulation were not provided, vaccinated birds were protected against challenge with both SE and ST [260].

Gallimune^®^ SE + ST (Boehringer Ingelheim Animal Health Netherlands B.V., 8221 RA Lelystad, The Netherlands) is an inactivated vaccine targeting SE and ST and is licensed for use in chickens. According to the manufacturer’s information, the vaccine should be administered IM as a primary vaccination, followed by a booster after four to ten weeks. There is currently no published evidence describing the use of this specific vaccine in non-domesticated bird species.

Nobilis^®^ Salenvac ETC (Intervet International B.V., 5831 AN Boxmeer, The Netherlands) is an inactivated vaccine directed against SE, ST, and *S. infantis*. The vaccine is licensed for use in chickens. The vaccine is administered IM, with the manufacturer recommending a booster dose at an interval of at least four weeks [261]. To date, no published studies have reported the use of this specific vaccine in non-domesticated bird species.

Salmovac^®^ (Pharmagal-Bio spol. s.r.o., 949 01 Nitra, Slovak Republic) is an inactivated SE and ST vaccine licensed for use in pigeons. According to the manufacturer’s leaflet, primary vaccinations may be performed from the age of four weeks, followed by a booster 21–28 days after the first vaccination. Adult birds should be administered an annual booster vaccination.

AviPro Salmonella vac E^®^ (Elanco, Utrecht, The Netherlands) and AviPro Salmonella DUO^®^ (Elanco, 3528 AE Utrecht, The Netherlands) are among the few commercial *Salmonella* vaccines for which efficacy data in poultry have been reported in the literature [262,263,264,265,266,267]. AviPro Salmonella DUO^®^ protects against SE and ST, whereas Vac E^®^ is specifically formulated to protect against SE. Both vaccines are administered orally, with primary vaccination followed by a second dose six to eight weeks later, and a third dose administered ten weeks thereafter. To date, no published studies have reported the use of these vaccines in avian species other than commercial poultry.

Poulvac^®^ ST (Zoetis Belgium S.A., 1930 Zaventem, Belgium) is a modified live vaccine that induces immunity against ST through a self-limiting infection. Primary vaccination is administered by spray, followed by a booster dose via drinking water two weeks later. To date, no published studies have described the use of this vaccine in avian species outside commercial poultry.

Several studies have evaluated *Salmonella* vaccination strategies in pigeons. In one study, an autogenous *Salmonella* vaccine induced sufficiently high antibody titers in 85% of birds vaccinated at 7 and 11 weeks of age, compared with 50% of birds vaccinated at 11 weeks only. However, the authors noted that vaccination does not prevent bacterial carriage or shedding [268]. Another study used two different inactivated *S. typhimurium* var. *Copenhagen* vaccines. A reduction in the level of fecal shedding and a less severe polydipsia were observed, but neither vaccine conferred protection against experimental challenge [269]. A third study evaluated a combined PPMV-1 and ST autogenous vaccine, which conferred 90% protection in vaccinated birds, as assessed by a hemagglutination assay [270]. Finally, a comparative study was conducted evaluating the immunological responses induced by an autogenous ST vaccine compared to Zoosal T^®^ (IDT Biomedika, 06861 Dessau-Rosslau, Germany), a commercial attenuated ST vaccine for pigeons. It was concluded that the humoral and cellular responses were twice as strong in the birds vaccinated with the autogenous vaccine [271].

### 4.5. Yersinia pseudotuberculosis

Infections and outbreaks of *Yersinia pseudotuberculosis* (Yp) have been reported in captive bird populations for decades, e.g., Bucerotiformes, Columbiformes, Musophagiformes, Passeriformes, Piciformes, and Psittaciformes. In birds, sudden death is the most common outcome. Clinical signs are often limited to lethargy, diarrhea, and anorexia or hyporexia [272].

A killed whole-cell vaccine is currently commercially available in Europe: Pseudovac^®^ (Department of Veterinary Pathology, Utrecht University, 3584 CS Utrecht, The Netherlands). Pseudovac^®^ contains serotypes I–VI isolates. It contains twenty different Yp strains isolated from outbreaks in different animal species and regions, which are updated annually. The manufacturer recommends administering Pseudovac^®^ SC, preferably late summer or in the autumn, since most of the reported outbreaks occur in wintertime and early spring [272]. The initial vaccination should be followed by a booster six weeks later, with another booster administered 12 months later. Although vaccine-induced protection declines after nine months, some European zoos administer a single annual vaccination prior to winter, as Yp is generally considered a minor concern during the summer and fall months in western Europe [272,273]. The recommended dosage is 0.25 mL SC for birds < 1 kg, 0.5 mL SC for birds weighing 1–5 kg, and 1.0 mL SC for birds > 5 kg body weight.

The production and administration of an autogenous vaccine must be based on isolates obtained from animals within the same epidemiological entity. Autogenous vaccines lack formal evidence of efficacy and safety in birds and, therefore, should be used with caution [274]. In two German zoos, an autologous vaccine was produced from a Yp isolate obtained from resident birds [275]. The isolates were cryopreserved, and a flock-specific vaccine was produced by Ceva BESTVAC (06861 Dessau-Rosslau, Germany). Following Yp-related mortality, this vaccine was administered twice to birds in their outdoor free-flight aviary at intervals of three to five weeks, with no adverse effects reported. Newly introduced birds received a primary immunization during quarantine. Despite this vaccination, two toco toucans (*Ramphastos toco*) died due to Yp infection.

One paper described the fabrication of stable vaccines that were administered yearly to the species that had been affected [276]. Further details are, unfortunately, not provided.

Following an outbreak associated with significant morbidity and mortality, an entire flock (*n* = 52) of village weavers (*Ploceus cucullatus*) was repeatedly vaccinated IM with an autogenous Yp vaccine [277]. An aluminum-adjuvanted, autogenous Yp vaccine was produced independently by two different laboratories (Nationwide Laboratories: www.nwlabs.co.uk (accessed on 25 December 2025), and Ridgeway Biologicals: www.ridgewaybiologicals.co.uk (accessed on 25 December 2025)). The laboratories used identical formulations, the same Yp isolates, and a crystalline aluminum hydroxide (boehmite) gel (Rehydragel; Chemtrade Logistics, www.chemtradelogistics.com (accessed on 25 December 2025)) as adjuvant. No immediate post-vaccination adverse reactions or yersiniosis-associated lesions were observed. However, between 74 and 408 days post-vaccination, three birds presented with impaired flight and abnormal wing carriage due to large, unilateral pectoral masses. Histological examination revealed anaplastic sarcomas containing intralesional crystalline material, suggesting presumptive vaccine-associated sarcomas (*n* = 3).

### 4.6. Pasteurella multocida

*Pasteurella multocida* is a zoonotic bacterium that may affect various animal species, causing hemorrhagic septicemia or infectious pneumonia [278,279]. *P. multocida* has been diagnosed in Galliformes, Psittaciformes, carrion crows (*Corvus corone corone*), rooks (*Corvus frugilegus*), yellow-eyed penguins (*Megadyptes antipodes*), crested auklets (*Aethia cristatella*), thick-billed murres (*Uria lomvia*), common eider (*Somateria mollissima*), northern fulmars (*Fulmarus glacialis*), gulls (*Larus* spp.), and turkeys [73,280,281,282,283].

An inactivated vaccine, Cevac^®^Landavax^®^SC (CEVA-Phylaxia Co. Ltd., 1107 Budapest, Hungary), is licensed for use in mulard ducks (a hybrid between domestic Muscovy ducks and domestic ducks). The recommended vaccination regimen consists of two SC doses of 0.5 mL administered into the lower back region of the neck at three weeks of age and again three weeks later. Cevac^®^Landavax^®^SC is commonly used off-label in layer chickens as well (Leus, pers. comm).

### 4.7. Avibacterium paragallinarum

Infectious coryza is caused by *Avibacterium paragallinarum* (formerly *Haemophilus paragallinarum*) and has been reported to cause disease in pheasants, guineafowl, Japanese quail, and gray crowned cranes (*Balearica regulorum*) [73,284]. Infectious coryza is characterized by inflammation of the upper respiratory tract, facial edema, conjunctivitis, nasal discharge, diarrhea, and anorexia. The wattles may also be affected.

Vaccination is possible; however, accurate identification of the causative bacterial serotype is essential [73]. In chickens, several studies have confirmed the safety and efficacy of autogenous vaccines against *A. paragallinarum*, *P. multocida*, *E. coli*, and ARV [285].

Coripravac^®^ (Hipra, 17170 Amer Girona, Spain) is a commercially available inactivated vaccine licensed for domestic poultry against *A. paragallinarum* serotypes A (strain 17756), B (strain 0222), and C (strain Modesto). In chickens, 0.5 mL should be administered SC or IM at the ages of 12 and 18 weeks. To date, no published studies have reported the use of this vaccine in non-domesticated bird species.

## 5. Fungal Etiology

### Aspergillus fumigatus

**Figure 6:**
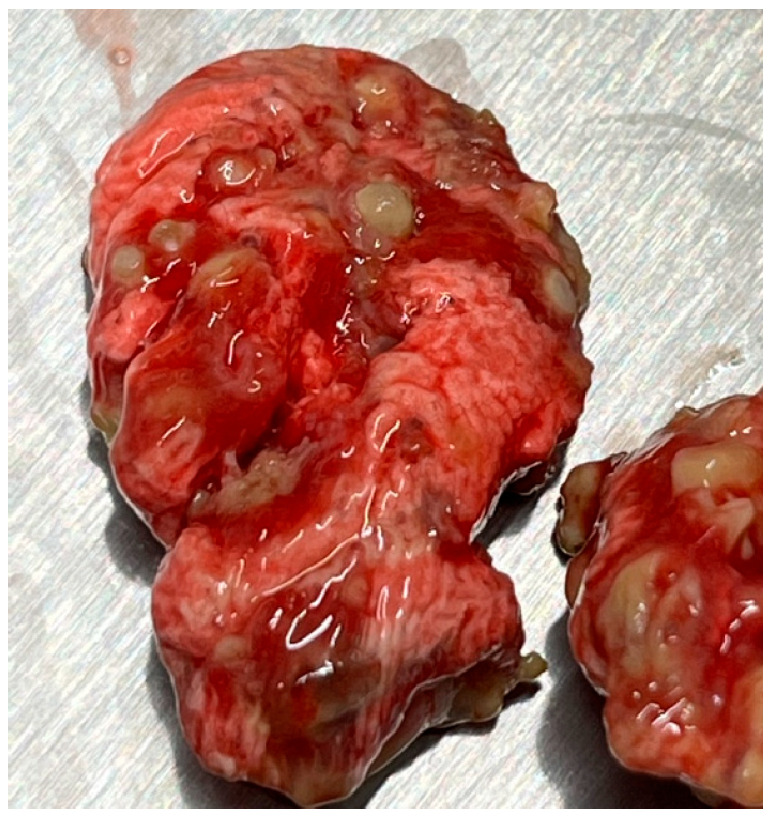
Lung severely affected by *Aspergillus fumigatus* in an Australian magpie (*Gymnorhina tibicen*) with multifocal mycotic granulomas (photograph provided by H. Kempf).

*Aspergillus fumigatus* is the primary causative agent of avian aspergillosis and is responsible for significant morbidity and mortality in a wide range of captive bird species [286,287,288]. Aspergillosis is a significant cause of mortality in captive penguins [289,290,291,292,293,294]. Other avian species that are commonly affected belong to the following taxonomic orders: Anseriformes, Accipitriformes, Charadriiformes, Passeriformes, and Galliformes [286,287,288]. Common reported clinical signs include gasping and deep, labored breathing. Ataxia may occur when the central nervous system is involved, and blindness can develop when infection localizes in the anterior chamber of the eye. On necropsy, yellow caseous plaques are commonly observed in the lungs, air sacs, trachea, and on peritoneal surfaces (Figure 6) [6].

Currently, no commercial vaccines are available to prevent aspergillosis; however, several experimental studies have been conducted.

In turkeys, poults were vaccinated with combinations of two different germling preparations and three adjuvants: N-acetylmuranyl-L-alanyl-D-isoglutamine, *P. multocida* lipopolysaccharide, and avridine. The animals were subsequently challenged with *A. fumigatus*. Results have been mixed, with variable immune responses reported [295].

The use of a whole-culture *A. fumigatus* vaccine was evaluated in tufted puffins (*Fratercula cirrhata*) [296]. Ten clinically healthy adult birds were vaccinated; however, two birds died from anaphylaxis within four hours of administration. Following a booster 23 weeks later, one bird died from acute aspergillosis and renal amyloidosis. Based on these findings, the authors concluded that this vaccine is not recommended for use in tufted puffins.

More promising results were obtained in a study evaluating vaccination in Inca terns (*Larosterna inca*) [297]. The Asp f3 vaccine, an antifungal formulation initially developed for human cancer patients, was used; this vaccine had previously demonstrated to confer protection against *A. fumigatus* in mice and chickens [298,299]. Inca tern chicks received three doses at two, four, and six weeks of age. Plasma analysis demonstrated the production of Asp f3-specific IgY titers after the first and second doses. Additionally, the incidence of aspergillosis-associated mortality decreased from 7.48% to 1.85%, with only one of the 54 (1.9%) vaccinated birds dying of aspergillosis between 2017 and 2019. Notably, this bird had received only two of the three scheduled doses [297].

In a study involving Humboldt penguins (*Spheniscus humboldti*) (*n* = 20), oral administration of a probiotic-based vaccine against aspergillosis was evaluated. The vaccine consisted of *E. coli* Nissle engineered to express high levels of α-Gal, with the aim of modulating the anti-α-Gal response. The vaccine was demonstrated to be safe and to confer a protective effect against aspergillosis. The authors concluded that this approach warrants further investigation as a potential preventative measure in birds [300].

## 6. Autogenous Vaccines

Autogenous vaccines are permitted for use only when vaccines with marketing authorization are unavailable in the EU, or when authorized vaccines cannot be supplied. The manufacture and use of autogenous vaccines are subject to specific national regulations and legislation that clearly distinguish them from conventional vaccines [301]. Following the EU legislation (Regulation EU 2019/6, Article 2 Nr 3), autogenous vaccines are defined as “inactivated immunological veterinary medicinal products which are manufactured from pathogens and antigens obtained from an animal or animals in an epidemiological unit and used for the treatment of that animal or those animals in the same epidemiological unit or for the treatment of an animal or animals in a unit having a confirmed epidemiological link”. Consequently, these vaccines are derived from a killed pathogen and are intended for use in a defined group of animals within a specific epidemiological setting.

Antigens derived from killed pathogens are combined with an adjuvant to enhance the immune response [301,302,303]. The most used adjuvants are based on mineral oil or aluminum hydroxide, each with distinct immunological properties. Autogenous vaccines formulated with aluminum hydroxide adjuvants generally confer protection for only a few weeks. However, the onset of immunity is relatively rapid, occurring approximately two weeks after administration [304,305]. The mineral-oil-based vaccines cause a strong local response at the administration site and induce a strong humoral response. This protection often lasts for months, but the onset of immunity is delayed up to four weeks [305].

**Figure 7:**
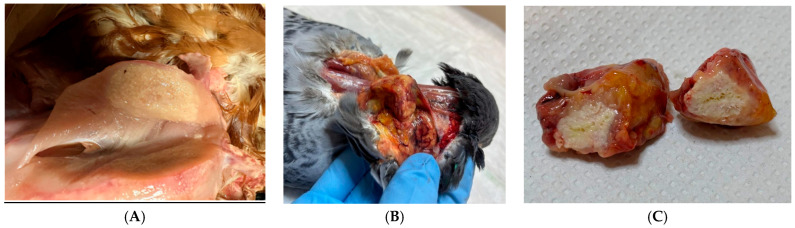
(**A**) Necrosis in the pectoral muscle of a laying hen due to an oil-based autogenous vaccine applied at a too low temperature; (**B**) in situ granulomatous reaction to an oil-based autogenous vaccine in the neck of a pigeon due to improper aseptic techniques during administration; (**C**) cross-section of a granulomatous reaction of an oil-based autogenous vaccine in the neck of a pigeon (photographs provided by J. Leus).

Adverse local effects at the administration site are frequently associated with both the route and conditions of administration. For example, administration of vaccines at excessively low temperatures may provoke marked local inflammatory reactions, potentially resulting in muscle necrosis (Figure 7A). Granulomatous reactions may occur following parental administration if aseptic techniques are not adequately maintained (Figure 7B,C) (Leus, pers. comm). Excessive local reactions to oil-based vaccines in pigeons have also been reported [306]. However, caution in the use of autogenous vaccines is warranted not only because of the risk of injection-site reactions, but also due to the potential for systemic and species-specific adverse effects.

An entire flock of village weavers was repeatedly vaccinated IM with an autogenous Yp vaccine [277]. Histological examination revealed anaplastic sarcomas containing intralesional crystalline material, suggesting presumptive vaccine-associated sarcomas (*n* = 3). Notably, this represents the first report of presumptive Yp vaccine-associated sarcoma in a non-mammalian species. This report highlights the need for caution when using autogenous vaccines with unconfirmed safety and effectiveness profiles.

Autogenous vaccines are commonly used in poultry and racing pigeons for both bacterial and viral pathogens. In chickens, studies have been performed on the safety and efficacy of autogenous vaccines for *A. paragallinarum*, *P. multocida*, *E. coli*, and ARV [168,200,201,202,285,307]. In ducks, studies have been conducted on autogenous vaccines for *Riemerella anatipestifer*, and in pigeons for *Streptococcus gallolyticus*, *S. typhimurium*, and PPMV-1 [268,270,271,308,309].

Autogenous vaccines have also been applied to other avian taxa for the management of specific pathogens, as discussed in Section 3.2.1, Section 3.2.4 and Section 4.5, and Section *Aspergillus fumigatus*.

## 7. One Health Implications

Birds may serve as natural reservoirs for numerous emerging viral pathogens and provide opportunities for the evolution, emergence, and spread of novel viruses [310]. Stress, overcrowding, human encroachment on wild bird habitats, domestication of wild birds as pets, and increasingly intensive poultry farming have facilitated the spillover of pathogens across species barriers, leading to zoonotic infections [1,113,252,311,312,313,314]. This issue is of particular concern in zoological institutions, where a high diversity of avian species often live in close proximity to local wildlife, animal caretakers, and, in some cases, zoo visitors [315,316,317]. The young, elderly, and immunocompromised are especially at risk [110,113,310,318,319,320,321]. Therefore, it is crucial to understand the complex interactions between birds, vectors, zoonotic pathogens, and the environment, particularly in the context of increasing urbanization and the emergence of zoonotic diseases [322]. Given the multifaceted nature of zoonotic pathogen transmission, effective surveillance and control require an integrated understanding of environmental conditions, host interactions, and pathogen adaptation processes [323]. In this context, HPAI viruses warrant particular attention, as ongoing viral evolution, widespread circulation in wild birds, spillover into domestic animals, and documented zoonotic infections highlight their potential to pose a future pandemic risk under favorable ecological and epidemiological conditions [324,325,326,327,328,329,330].

## 8. Discussion

Vaccination constitutes an important preventive tool in the health management of birds housed in zoological collections, yet it presents substantial clinical and logistical challenges. Although a wide range of commercial vaccines is available for domestic poultry, their off-label use in non-domesticated species is often guided by anecdotal experience rather than supported by peer-reviewed safety and efficacy studies. In the absence of species-specific protocols, clinicians must rely on recommended vaccination schedules for poultry to prevent disease in captive non-domesticated birds. Nevertheless, caution should always be exercised when first using a vaccine off-label in a novel species, as vaccine safety and effectiveness may vary considerably, even between closely related taxa.

Interpretation of vaccination outcomes in non-domesticated avian species is constrained by several structural and methodological limitations inherent to this field. Ethical considerations and animal welfare regulations restrict the use of experimental challenge studies, while the taxonomic diversity of avian species and the small size of managed populations limit the feasibility of controlled trials with sufficient statistical power. As a result, much of the available evidence is derived from poultry vaccination studies, serological response data, or observational field reports rather than direct measures of clinical protection. These constraints necessitate cautious extrapolation of vaccine efficacy and underscore the need to interpret immunogenicity endpoints in the context of their known limitations [8,9,10].

Autogenous vaccines may offer valuable, targeted protection during outbreaks, high-risk periods, or in areas with high infection pressure. However, their application should be approached cautiously due to the risks of injection-site reactions, systemic adverse effects, and species-specific responses. Moreover, data regarding their safety profile and duration of immunity in non-domesticated avian species remain limited.

Vaccination challenges are compounded by the husbandry conditions typical of zoological institutions. Compared with domestic poultry, many non-domesticated bird species are more sensitive to stress and are often housed in free-flight aviaries, often rendering capture and parenteral vaccination difficult. In such contexts, non-injectable delivery methods, such as oral administration via drinking water vaccines, may offer practical advantages if efficacy can be ensured. This conflicts with the preference for inactivated vaccines over live-attenuated vaccines in off-label species due to safety concerns, as attenuated vaccines are generally not available for non-invasive administration routes.

Additionally, the conditions under which commercial poultry vaccines are developed and applied differ substantially from those encountered in zoological settings. Commercial poultry production typically relies on all-in–all-out management systems, strict separation between generations, and extensive disinfection protocols. In contrast, zoological collections often maintain multigenerational avian groups within the same enclosure, increasing the likelihood of early and repeated exposure to pathogens or vectors. Consequently, initiation of vaccination at a later age in zoo settings may occur after animals have already been exposed to infectious agents, potentially limiting vaccine effectiveness.

Beyond vaccination, it is important to recognize that global pathogen dissemination is driven not only by avian migration but also by the intersection of wildlife trade and transport, intensive farming systems, and human travel. These factors collectively increase the risk of pathogen emergence and pandemic spread. Therefore, vaccination should be integrated into a broader preventive framework that includes routine pathogen surveillance, diagnostic testing, and strict quarantine and biosecurity measures to limit transmission among susceptible hosts.

While vaccination may play a pivotal role in disease prevention, advancing evidence-based immunization programs will depend on improved reporting of outcomes, collaborative research efforts, and the development of species-specific safety and efficacy studies. Strengthening this knowledge base is essential to improving health, welfare, and conservation outcomes for avian populations maintained in zoological collections worldwide.

## 9. Conclusions

Given the acknowledged lack of validated vaccines and limited efficacy data, vaccination should be framed as a potential tool only in carefully selected contexts. At present, no harmonized European recommendations exist regarding vaccination of avian species housed in zoological collections, and vaccination practices vary widely depending on national legislation, regulatory oversight, institutional policies, and case-by-case risk assessments, as well as species-specific considerations and post-vaccination monitoring strategies. An overview of the available vaccines per viral (Table S1) and bacterial (Table S2) pathogens is provided.

## Data Availability

No new data were created or analyzed in this study. Data sharing is not applicable to this article.

## Supplementary Materials

The following supporting information can be downloaded at: https://www.mdpi.com/article/10.3390/vetsci13020148/s1, Table S1: Overview of the available vaccines per virus per avian species; Table S2: Overview of the available vaccines per bacteria per avian species.
