# Supplementary material for: An Overview of Avian Vaccination Options in Zoological Collections in Europe
Source: Vet Sci. 2026 Feb 4;13(2):148. doi: 10.3390/vetsci13020148 (PMC12945050; doi:10.3390/vetsci13020148)
Supplement: Supplementary file 1 [file vetsci-13-00148-s001.zip › vetsci-4103148-supplementary.pdf]

Table S1. Overview of the available vaccines per virus per avian species. When indicated in red, vaccination is not recommended for at least one species in the taxonomic order/family/genus
*Leus, pers. comm
** Officially registered in at least one country, for at least one species in the taxonomic order/family/genus (except from chickens)
***Kempf, pers. comm
****Baumgartner, pers. comm
When indicated in red, vaccination is not recommended for at least one species in the taxonomic order/family/genus

	Avipoxvirus	Infectious laryngotracheitis virus	Marek disease	Pacheco disease	Columbid Herpesvirus	Duck Viral enteritis	Avian Influenza	Newcastle Disease	Parrot Bornavirus	Polyomavirus	Psittacine beak and feather disease	West Nile Virus	Eastern equine encephalomyelitis and Western equine encephalomyelitis virus	Avian Reovirus	Adenovirus	Infectious Bronchitis Virus	Avian metapneumovirus	Parvovirus
Galliformes	Nobilis®AE+PD*	Innovax®-ILT-IBD*,**<br>Attenuated ILT vaccines[62,73]	Nobilis®Rismavac*,**<br>Innovax®-ILT-IBD*,**<br>Innovax®-ND-IBD*,**<br>Poulvac® Marek CVI+HVT, ,**	/	/	/	Inactivated H5N9 vaccine[13,91]<br>Inactivated H5N2 vaccine[90]<br>Nobilis® Influenza H5N2[93]<br>RNA replicon vaccine[89]	Avishield®ND B1*,**<br>Nobilis®RT + IBmulti + ND + EDS*,**<br>Nobilis®Ma5 + Clone 30**,***<br>Innovax®-ND-IBD*,**	/	/	/	EQUIP WNV®[12]<br>West Nile-Innovator®[16]	/	/	Attenuated vaccines[171,172]<br>Dindoral®SPF**	Nobilis®RT + IBmulti + ND + EDS*,**<br>Nobilis®Ma5 + Clone 30**,***<br>Poulvac®IB QX*,**<br>Poulvac®IB Primer*,**	Nobilis®RT + IBmulti + ND + EDS*,**<br>Nobilis®TRT[194]	/
Anseriformes	Nobilis®AE+PD*<br>Diftosec®*	Nobilis®-ILT-IBD*	Nobilis®Rismavac*<br>Nobilis®-ILT-IBD*<br>Innovax®-ND-IBD*	/	/	Kapevac®**<br>Vaxiduk®**	Inactivated H5N9 vaccine[13,91]<br>Inactivated H5N2 vaccine[90]<br>Nobilis® Influenza H5N2[93]<br>RNA replicon vaccine[89]	Innovax®-ND-IBD*	/	/	/	EQUIP WNV®[12]	/	/	/		No vaccine tried	Deparmune®**
Columbiformes	Diftovac**	/	/	/	Pharmavac Columbi 2®**<br>Pharmavac PHA®**	/	Inactivated H5N9 vaccine[91]<br>Inactivated H5N2 vaccine[90]	Pharmavac Columbi 2®**<br>Pharmavac PHA®**<br>RP Vacc®**<br>Nobilis®Paramyxo P201**<br>Colombovac®PMV	/	/	/	/	/	/	Pharmavac PHA®**			/
Psittaciformes	Experimental vaccine[40]	/	/	Autogenous vaccine[47,49]	/	/	Inactivated H5N9 vaccine[13,91]<br>Inactivated H5N2 vaccine[90]<br>Nobilis® Influenza H5N2[93]	Attenuated Hitchner B1, La Sota and an inactivated vaccine[115]	Experimental vaccine[124-127]	Experimental vaccines[130-133]<br>Psittimune®APV**	Experimental vaccines[139,140]	EQUIP WNV®[12]	/	/	/			/
Struthiformes	Commercial FP vaccine[26]	/	/	/	/	/	Inactivated H5N9 vaccine[13,91]<br>Inactivated H5N2 vaccine[90]<br>Nobilis® Influenza H5N2[93]	Pestikal®[102]	/	/	/	EQUIP WNV®[12]	Equiloid®[158]<br>Triple-E®[158]					
Otidiformes	Houbarapox vaccine®**<br>Poulvac® P Canary[34]	/	/	/	/	/	/	Attenuated Hitchner B1 and Nobilis®Newcavac[114,116]	/	/	/	/	/	Nobilis®Reo Inac[33]	/			/
Gruiformes	/	/	No vaccine tried	/		/	Inactivated H5N9 vaccine[13,91]<br>Inactivated H5N2 vaccine[90]	/	/	/	/	EQUIP WNV®[12]	Human-licensed EEE vaccine[162]	/	/			/
Strigiformes	/	/	/	/	No vaccine tried	/	Inactivated H5N9 vaccine[91]<br>Inactivated H5N2 vaccine[90]<br>Nobilis® Influenza H5N2[93]<br>RNA replicon vaccine[89]	No vaccine tried	/	/	/	EQUIP WNV®[12]	/	/	/			/
Accipitriformes	/	/	/	/	No vaccine tried	/	Inactivated H5N9 vaccine[91]	No vaccine tried	/	/	/	EQUIP WNV®[12]<br>Experimental DNA vaccine[155]	/	/	/			/
Falconiformes	Falconpox Vaccine®[28-32]	/	/	/	Experimental attenuated vaccine[83]	/	Inactivated H5N9 vaccine[91]<br>Inactivated H5N2 vaccine[90]<br>Nobilis® Influenza H5N2[93]	Inactivated vaccine[29-31,114]	/	/	/	EQUIP WNV®[12]<br>Recombitek®[153]<br>Duvaxyn®[153]	/	/	/			/
Passeriformes	Poulvac® P Canary**<br>Biomune Poximune C®[38],**	/	/	/	/	/	Inactivated H5N9 vaccine[91]<br>Inactivated H5N2 vaccine[90]<br>Nobilis® Influenza H5N2[93]<br>RNA replicon vaccine[89]	/	Experimental vaccine[126]	/	/	EQUIP WNV®[12]<br>West Nile-Innovator®<br>Recombitek®	No vaccine tried	/	/			/
Bucerotiformes	/	/	/	/	/	/	Nobilis® Influenza H5N2[93]	Attenuated vaccine and Struvac®[104]	/	/	/		/	/	/			/
Sphenisciformes	/	/	/	/	/	/	Inactivated H5N9 vaccine[91]<br>Inactivated H5N2 vaccine[90]<br>Nobilis® Influenza H5N2[93]<br>RNA replicon vaccine[89]	No vaccine tried	/	/	/	Inactivated vaccine<br>DNA vaccine[145]<br>Innovator®[146]	/	/	/			/
Musophagiformes	/	/	/	/	/	/	Inactivated H5N2 vaccine[90]	Attenuated Hitchner B1, La Sota and an inactivated vaccine[115]	/	/	/	/	/	/	/			/
Coraciiformes							Inactivated H5N9 vaccine[13,91]<br>Nobilis® Influenza H5N2[93]					EQUIP WNV®[12]	/					
Phoenicopteriformes							Inactivated H5N9 vaccine[13,91]<br>Inactivated H5N2 vaccine[90]<br>Nobilis® Influenza H5N2[93]<br>RNA replicon vaccine[89]					EQUIP WNV®[12]<br>Innovator®[146]	/					
Pelecaniformes							Inactivated H5N9 vaccine[13,91]<br>Inactivated H5N2 vaccine[90]<br>Nobilis® Influenza H5N2[93]<br>RNA replicon vaccine[89]					EQUIP WNV®[12]	/					
Charadriiformes							Inactivated H5N9 vaccine[13,91]<br>Inactivated H5N2 vaccine[90]<br>Nobilis® Influenza H5N2[93]											
Ciconiiformes							Inactivated H5N9 vaccine[91]<br>Inactivated H5N2 vaccine[90]<br>Nobilis® Influenza H5N2[93]<br>RNA replicon vaccine[89]											
Cuculiformes							Inactivated H5N2 vaccine[90]											
Piciformes							Inactivated H5N9 vaccine[91]											

Table S2. Overview of the available vaccines per bacteria per avian species. When indicated in red, vaccination is not recommended for at least one species in the taxonomic order/family/genus
*Leus, pers. comm
** Officially registered in at least one country, for at least one species in the taxonomic order/family/genus (except from chickens)
***Kempf, pers. comm
****Baumgartner, pers. comm

	Mycoplasma gallisepticum	Erysipelothrix rhusiopathiae	Salmonella spp.	Yersinia pseudotuberculosis	Pasteurella multocida	Avibacterium paragallinarum	Aspergillus fumigatus	Riemerella anatipestifer	Streptococcus gallolyticus
Galliformes	FVAXX-MG®<br>Poulvac®MG*	Eryvac®[227]	Inactivated vaccine[260,261]	/	No vaccine tried	No vaccine tried	No vaccine tried	/	/
Anseriformes	No vaccine tried	No vaccine tried	No vaccine tried	/	Cevac®Landavax®SC*	/	No vaccine tried	Autogenous vaccine[308]	/
Columbiformes	/	/	Inactivated vaccine[269,271]<br>Autogenous vaccine[268,270,271]	No vaccine tried	/	/	/	/	Autogenous vaccine[309]
Psittaciformes	No vaccine tried	Inactivated bacterin[232,233]	No vaccine tried	No vaccine tried	No vaccine tried	/	/	/	/
Struthiformes	/	Vaxall Erysipelas vaccine®[236]	/	/	/	/	/	/	/
Otidiformes	/	/	/	/	/	/	/	/	/
Gruiformes	/	/	/	/	/	No vaccine tried	/	/	/
Strigiformes	/	/	/	/	/	/	/	/	/
Accipitriformes	/	No vaccine tried	/	/	/	/	No vaccine tried	/	/
Falconiformes	No vaccine tried	/	/	/	/	/	/	/	/
Passeriformes	No vaccine tried	/	No vaccine tried	Autogenous vaccine[277]	No vaccine tried	/	No vaccine tried	/	/
Bucerotiformes	/	/	/	Pseudovac®[273]	/	/	/	/	/
Sphenisciformes	/	No vaccine tried	No vaccine tried	/	No vaccine tried	/	Probiotic-based vaccine[300]	/	/
Musophagiformes	/	/	/	No vaccine tried	/	/	/	/	/
Coraciiformes	/	No vaccine tried	/	/	/	/	/	/	/
Phoenicopteriformes	No vaccine tried	/	/	Autogenous vaccine[275]	/	/	/	/	/
Pelecaniformes	/	Nobilis®Erysipelas[234]	/	/	/	/	/	/	/
Charadriiformes	/	Nobilis®Erysipelas[234]	/	/	/	/	Autogenous vaccine[296]<br>Asp f3 vaccine[297]	/	/
Ciconiiformes	/	/	/	/	/	/	/	/	/
Cuculiformes	/	/	/	/	/	/	/	/	/
Piciformes	/	/	/	Autogenous vaccine[275]	/	/	/	/	/
